# Evaluation and mitigation of the limitations of large language models in clinical decision-making

**DOI:** 10.1038/s41591-024-03097-1

**Published:** 2024-07-04

**Authors:** Paul Hager, Friederike Jungmann, Robbie Holland, Kunal Bhagat, Inga Hubrecht, Manuel Knauer, Jakob Vielhauer, Marcus Makowski, Rickmer Braren, Georgios Kaissis, Daniel Rueckert

**Affiliations:** 1 Institute for AI and Informatics, Klinikum rechts der Isar, Technical University of Munich, Munich, Germany; 2 Institute for Diagnostic and Interventional Radiology, Klinikum rechts der Isar, Technical University of Munich, Munich, Germany; 3 Department of Computing, Imperial College, London, UK; 4 Department of Medicine, ChristianaCare Health System, Wilmington, DE USA; 5 Department of Medicine III, Klinikum rechts der Isar, Technical University of Munich, Munich, Germany; 6 Department of Medicine II, University Hospital of the Ludwig Maximilian University of Munich, Munich, Germany; 7 Reliable AI Group, Institute for Machine Learning in Biomedical Imaging, Helmholtz Munich, Munich, Germany

**Keywords:** Diagnosis, Health care economics, Translational research

## Abstract

Clinical decision-making is one of the most impactful parts of a physician’s responsibilities and stands to benefit greatly from artificial intelligence solutions and large language models (LLMs) in particular. However, while LLMs have achieved excellent performance on medical licensing exams, these tests fail to assess many skills necessary for deployment in a realistic clinical decision-making environment, including gathering information, adhering to guidelines, and integrating into clinical workflows. Here we have created a curated dataset based on the Medical Information Mart for Intensive Care database spanning 2,400 real patient cases and four common abdominal pathologies as well as a framework to simulate a realistic clinical setting. We show that current state-of-the-art LLMs do not accurately diagnose patients across all pathologies (performing significantly worse than physicians), follow neither diagnostic nor treatment guidelines, and cannot interpret laboratory results, thus posing a serious risk to the health of patients. Furthermore, we move beyond diagnostic accuracy and demonstrate that they cannot be easily integrated into existing workflows because they often fail to follow instructions and are sensitive to both the quantity and order of information. Overall, our analysis reveals that LLMs are currently not ready for autonomous clinical decision-making while providing a dataset and framework to guide future studies.

## Abstract (web summary)

Using a curated dataset of 2,400 cases and a framework to simulate a realistic clinical setting, current large language models are shown to incur substantial pitfalls when used for autonomous clinical decision-making.

## Main

Large language models (LLMs) have the potential to revolutionize our medical system^1^ having shown their capabilities on diverse tasks^2–11^. Importantly, as humans primarily interact with the world through language, LLMs are poised to be the point of access to the multimodal medical artificial intelligence (AI) solutions of the future^12^. Until now, however, the diagnostic capabilities of models have been tested in structurally simple medical contexts, such as canonical vignettes of hypothetical patients or clinical case challenges. In both scenarios, all the required diagnostic information is provided upfront, and there is a single answer to be selected from a list of options. This type of question dominates both medical licensing exams^13–16^, where LLMs score well above passing^8–10,17–20^, and clinical case challenges, where models rival clinician performance^21–24^.

However, while these medical licensing exams and clinical case challenges are suitable for testing the general medical knowledge of the test-taker, they are far removed from the daily and complex task of clinical decision-making. It is a multistep process that requires gathering and synthesizing data from diverse sources and continuously evaluating the facts to reach an evidence-based decision on a patient’s diagnosis and treatment^25,26^. As this process is very labor intensive, great potential exists in harnessing AI, such as LLMs, to alleviate much of the workload. LLMs can summarize reports^3–5^, generate reports^2,4^, serve as diagnostic assistants^21,27^ and could ultimately autonomously diagnose patients. To understand how useful LLMs would be in such an autonomous, real-world setting, they must be evaluated on real-world data and under realistic conditions. However, the only analysis that tested an LLM throughout the diagnostic clinical workflow used curated lists of possible answers and examined only 36 hypothetical clinical vignettes^28^. Furthermore, any model that is used in such a high-stakes clinical context must not only be highly accurate, but also adhere to diagnostic and treatment guidelines, be robust, and follow instructions, all of which have not been tested in previous medical evaluations.

Here, we present a curated dataset based on the Medical Information Mart for Intensive Care (MIMIC-IV) database spanning 2,400 real patient cases and 4 common abdominal pathologies (appendicitis, pancreatitis, cholecystitis and diverticulitis) as well as a comprehensive evaluation framework around our dataset to simulate a realistic clinical setting. We provide LLMs with a patient’s history of present illness and ask them to iteratively gather and synthesize additional information such as physical examinations, laboratory results and imaging reports until they are confident enough to provide a diagnosis and treatment plan. Our dataset, task and analysis comprise a large-scale evaluation of LLMs on everyday clinical decision-making tasks in a realistic, open-ended environment. Unlike previous works, we test the autonomous information-gathering and open-ended diagnostic capabilities of models, representing an essential step toward evaluating their suitability as clinical decision-makers.

To understand how useful LLMs would be as second readers, we compare the diagnostic accuracy of the models with that of clinicians. Furthermore, we propose and evaluate a range of characteristics beyond diagnostic accuracy, such as adherence to diagnostic and treatment guidelines, correct interpretation of laboratory test results, instruction-following capabilities, and robustness to changes in instructions, information order and information quantity. Finally, we show that summarizing progress and filtering laboratory results for only abnormal results addresses some of the current limitations of models. We make our evaluation framework and dataset freely and openly available to guide future studies considering the use of LLMs in clinical practice.

## Results

### Creating the MIMIC-CDM dataset and evaluation framework

**Fig. 1:**
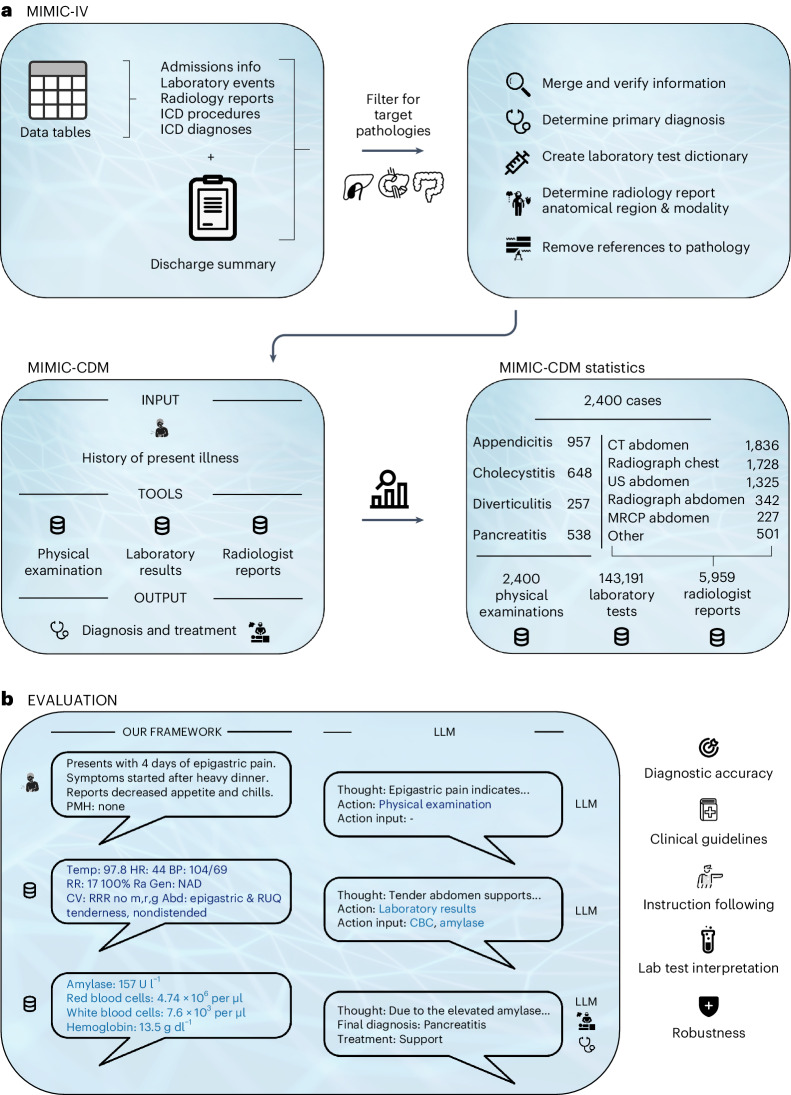
Overview of dataset creation and evaluation framework. **a**, To properly evaluate LLMs for clinical decision-making in realistic conditions, we created a curated dataset from real-world cases derived from the MIMIC-IV database, which contains comprehensive electronic health record data recorded during hospital admissions. **b**, Our evaluation framework reflects a realistic clinical setting and thoroughly evaluates LLMs across multiple criteria, including diagnostic accuracy, adherence to diagnostic and treatment guidelines, consistency in following instructions, ability to interpret laboratory results, and robustness to changes in instruction, information quantity and information order. ICD, International Classification of Diseases; CT, computed tomography; US, ultrasound; MRCP, magnetic resonance cholangiopancreatography.

Our curated dataset, MIMIC-IV-Ext Clinical Decision Making (MIMIC-CDM), is created using the well-established MIMIC-IV database, which contains de-identified electronic health records^29^. Figure 1a and ‘MIMIC-CDM dataset’ in Methods list the steps involved in creating the MIMIC-CDM dataset and its makeup. Our dataset contains data from 2,400 unique patients presenting with acute abdominal pain to the emergency department and whose primary diagnosis was one of the following pathologies: appendicitis, cholecystitis, diverticulitis or pancreatitis. We chose these target pathologies as they represent clinically important diagnoses of a common chief complaint, abdominal pain, which accounts for 10% of all emergency department visits^30,31^. Importantly, good differentiation between the four pathologies can be achieved using standard diagnostic tests, all of which are present in our dataset.

To reflect a realistic clinical setting that allows LLMs to autonomously engage in every step of the clinical decision-making process, we have created a comprehensive evaluation framework around our dataset. Using our framework and dataset, we present LLMs with a patient’s history of present illness and task them to gather and synthesize information to arrive at a diagnosis and treatment plan, which we evaluate for diagnostic accuracy as well as adherence to guidelines, as shown in Fig. 1b and explained in ‘Evaluation framework’ in Methods. For comparisons with practicing clinicians and further tests concerning robustness, we evaluate the diagnostic accuracy of LLMs as second readers, providing all necessary information for a diagnosis upfront, which we call MIMIC-IV-Ext Clinical Decision Making with Full Information (MIMIC-CDM-FI).

In our study, we tested the leading open-access LLM developed by Meta, Llama 2 (ref. ^32^), and its derivatives. We test both generalist versions such as Llama 2 Chat (70B)^32^, Open Assistant (OASST) (70B)^33^ and WizardLM (70B)^34^, as well as medical-domain aligned models such as Clinical Camel (70B)^19^ and Meditron (70B)^35^. Further information on the models and our selection criteria can be found in ‘Models’ in Methods and Table 1. Data taken from the MIMIC database is currently prohibited from being used with external application programming interfaces (APIs), such as that of OpenAI or Google, due to data privacy concerns and data usage agreements, so neither Chat-GPT, GPT-4, nor Med-PaLM could be tested. We note that Llama 2, Clinical Camel and Meditron have been shown to match and even exceed Chat-GPT performance on medical licensing exams and biomedical question answering tests^19,35^.

### LLMs diagnose significantly worse than clinicians

To ensure the patient’s safety in an autonomous clinical decision-making scenario, LLMs must diagnose at least as well as clinicians. Thus, we compared the diagnostic accuracy of the models on a subset of 80 patients of MIMIC-CDM-FI to four hospitalists with varying degrees of experience and from two countries. The makeup of the subset and details of the reader study can be found in ‘Reader study’ in Methods.

**Fig. 2:**
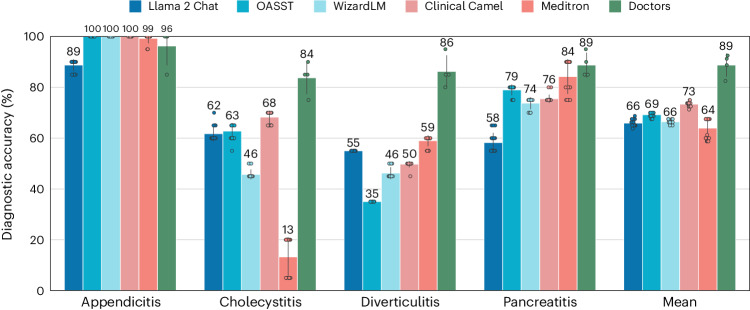
LLMs diagnose significantly worse than doctors when provided with all information. On a subset (*n* = 80) of MIMIC-CDM-FI, we compared the mean diagnostic accuracy of LLMs over multiple seeds (*n* = 20) with clinicians (*n* = 4) and found that LLMs perform significantly worse on average (*P* < 0.001) and especially on cholecystitis (*P* < 0.001) and diverticulitis (*P* < 0.001). The mean diagnostic accuracy is shown above each bar. Vertical lines indicate the standard deviation. The individual data points are shown.

We find that current LLMs perform significantly worse than clinicians on aggregate across all diseases (doctors versus Llama 2 Chat, *P* < 0.001; doctors versus OASST, *P* < 0.001; doctors versus WizardLM, *P* < 0.001; doctors versus Clinical Camel, *P* < 0.001; doctors versus Meditron, *P* < 0.001; Fig. 2). The difference in mean diagnostic performance between doctors and models was also large, ranging from 16 to 25 points. The diagnostic accuracy between the clinicians varied, with the German hospitalists in residency (mean = 87.50% ± 3.68%) performing slightly worse than the more senior US hospitalist (mean = 92.50%), which could be attributed to differences in experience and language and differing guidelines between the countries.

Most models were able to match clinician performance on the simplest diagnosis, appendicitis, where 3 of 4 clinicians also correctly diagnosed 20 of 20 patients. While the Meditron model matched or exceeded the other models at diagnosing patients with appendicitis, diverticulitis and pancreatitis, it failed for cholecystitis, diagnosing most patients simply with ‘gallstones’ without mention of inflammatory effects. This mirrors the general performance of the models, which may perform well on certain pathologies but currently lack the diagnostic range of human hospitalists. In a standard clinical scenario, where every diagnosis is a possibility, models must perform consistently across all pathologies of a single initial complaint, such as abdominal pain, to be useful.

Neither of the two specialist models performed significantly better on aggregate across all diseases and models (Clinical Camel versus Llama 2 Chat, *P* = 0.01; Clinical Camel versus OASST, *P* = 0.65; Clinical Camel versus WizardLM, *P* = 0.10; Meditron versus Llama 2 Chat, *P* > 1; Meditron versus OASST, *P* = 0.76; Meditron versus WizardLM, *P* > 1; Fig. 2). As the medical LLMs are not instruction tuned (that is, trained to understand and undertake new tasks), they are unable to complete the full clinical decision-making task where they must first gather information and then come to a diagnosis. As this is the primary-use case of a clinical decision-making model, we excluded them from all further analysis and only examined the Llama 2 Chat, OASST and WizardLM models for the rest of this work.

**Fig. 3:**
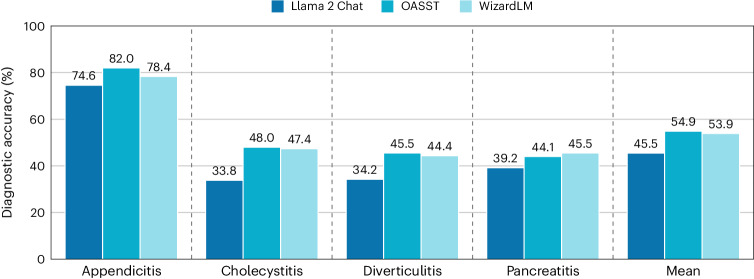
Diagnostic accuracy of LLMs decreased in an autonomous clinical decision-making scenario. When tasked with gathering all information required for clinical decision-making themselves, LLMs perform best when diagnosing appendicitis but perform poorly on the other three pathologies of cholecystitis, diverticulitis and pancreatitis. In such a realistic clinical scenario, model performance decreased compared to the retrospective diagnosis with all information provided (MIMIC-CDM-FI). The exact diagnostic accuracy is shown above each bar.

**Extended Data Fig. 1:**
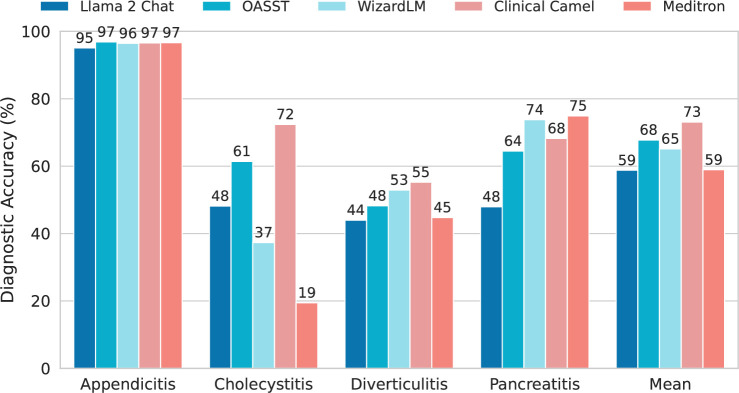
Model performance on the MIMIC-CDM-FI dataset. LLMs perform best on the MIMIC-CDM-FI dataset where all information required for a diagnosis is provided, especially on pathologies with strong indications such as appendicitis (dilated appendix described in radiologist report) and pancreatitis (elevated pancreatic enzymes listed in laboratory test results).

In our simulated clinical environment, which uses the MIMIC-CDM dataset, the LLM must specify all information it wishes to gather to accurately diagnose a patient. We observed a general decrease in performance, compared to MIMIC-CDM-FI (Extended Data Fig. 1), across all pathologies (Fig. 3). The mean diagnostic averages fell to 45.5% (versus 58.8% on MIMIC-CDM-FI) for Llama 2 Chat, 54.9% (versus 67.8%) for OASST and 53.9% (versus 65.1%) for WizardLM. All models performed best in diagnosing appendicitis (Llama 2 Chat, 74.6%; OASST, 82.0%; WizardLM, 78.4%), which is most likely because patients with appendicitis have consistent key symptoms with 791 of 957 radiologist reports (82.7%) directly stating that the appendix is dilated, enlarged or filled with fluid, and typically lack other intra-abdominal pathology descriptions that distract from the acute diagnosis.

In summary, LLMs do not reach the diagnostic accuracy of clinicians across all pathologies when functioning as second readers, and degrade further in performance when they must gather all information themselves. Thus, without extensive physician supervision, they would reduce the quality of care that patients receive and are currently unfit for the task of autonomous clinical decision-making.

### Current LLMs are hasty and unsafe clinical decision-makers

In addition to poor diagnostic accuracy, LLMs often fail to order the exams required by diagnostic guidelines, do not follow treatment guidelines and are incapable of interpreting lab results, making them a risk to patient safety. The current clinical guidelines used for this study are available in the literature for appendicitis^36^, cholecystitis^37^, diverticulitis^38^ and pancreatitis^39^.

**Extended Data Fig. 2:**
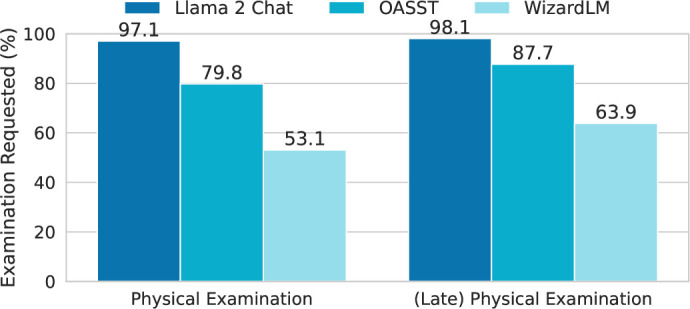
LLMs fail to consistently ask for a physical examination. The diagnostic guidelines of each disease require a physical examination to be performed as the first action. Llama 2 Chat was the only LLM that consistently requested physical examinations. (Late) Physical Examinations counted the physical examination if it wasn’t the first information requested.

All guidelines recommended physical examinations as an essential part of the diagnostic process, preferably as the first action. We find that only Llama 2 Chat consistently asks for physical examination results, either as the first action (97.1%) or at all (98.1%; Extended Data Fig. 2). The other two models requested less examinations (OASST, 79.8% and 87.7%; WizardLM, 53.1% and 63.9%), thereby omitting an essential piece of information.

**Extended Data Fig. 3:**
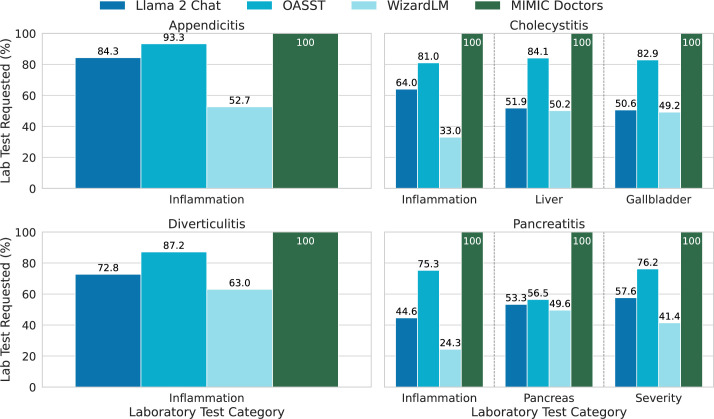
LLMs often do not order the necessary laboratory tests required to establish a diagnosis. The tests, defined by current diagnostic guidelines, help differentiate abdominal pathologies, as results can indicate which organ is currently pathologically afflicted or functioning normally. When testing the presence of the necessary tests in the MIMIC-CDM dataset, we find that the MIMIC Doctors requested all necessary tests in every patient case.

Based on the diagnostic guidelines, we defined categories of necessary laboratory tests for each pathology, including signs of inflammation, functional fitness of the liver and gallbladder, pancreas enzymes, and the severity of a patient’s pancreatitis. For our evaluation, we expect at least one test from each category to be requested, and the exact tests included in each category can be found in Supplementary Section A. We found that no model consistently orders all necessary categories, despite each test category being independently requested by all doctors in the MIMIC-CDM dataset (Extended Data Fig. 3). While OASST performs better than the other two models, reaching up to 93.3% and 87.2% in the inflammation category for appendicitis and diverticulitis, it often does not order the necessary tests for a diagnosis of pancreatitis (pancreas enzymes, 56.5%; severity, 76.2%), partially explaining why its diagnostic performance on pancreatitis was only 44.1% (Fig. 3).

**Extended Data Fig. 4:**
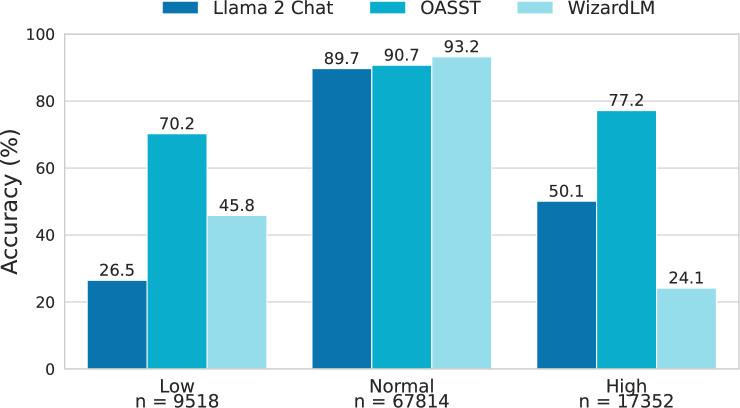
LLMs are incapable of interpreting lab results. To test the ability of LLMs to interpret laboratory data, we provided each laboratory test result and its reference range and asked the model to classify the result as below the reference range (low), within the range (normal) or above the range (high). We found that LLMs are incapable of consistently interpreting the result as normal, low or high, despite being provided with all required information. The models performed especially poorly on abnormal results which are of particular importance to establishing a diagnosis.

While it is important to order the correct laboratory tests, it is even more essential to correctly interpret them. To test the interpretation capabilities of the models, we provided each test result with the accompanying reference range and asked them to classify each result as either below, within or above the provided range. Any human with numerical literacy should be able to achieve perfect accuracy on such a task; however, all LLMs performed very poorly, especially in the critical categories of low test results (Chat, 26.5%; OASST, 70.2%; WizardLM, 45.8%) and high test results (Chat, 50.1%; OASST, 77.2%; WizardLM, 24.1%; Extended Data Fig. 4). Such a basic incomprehension of laboratory test results is a great risk to patient safety and must be resolved before LLMs become useful in a diagnostic capacity.

**Extended Data Fig. 5:**
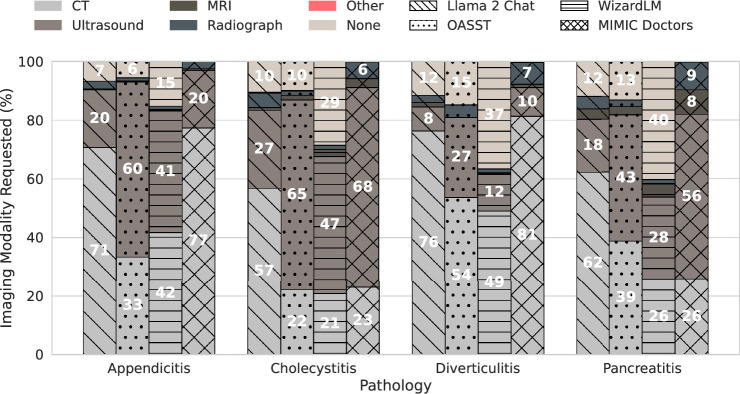
The first imaging modality requested by the LLMs and the doctors in the MIMIC dataset. LLMs sometimes follow diagnostic guidelines concerning imaging but often diagnose without requesting any imaging at all. As we show that imaging is the most useful diagnostic tool for all LLMs for each pathology except pancreatitis, this could be partly responsible for their low diagnostic accuracy. The legend specifies the colors of the imaging modalities and the patterns of the models.

While diagnostic guidelines provide advice on the potential use of imaging, highlighting the strengths and weaknesses of each modality in the context of the disease and the patient’s condition, the use of imaging in clinical practice can vary. We found that models sometimes matched the modalities requested by the doctors in the dataset, but often came to a diagnosis without requesting an abdominal imaging scan (Extended Data Fig. 5). We do not explicitly penalize models for not requesting an imaging scan, but as we later show that imaging is the most useful diagnostic tool for the LLMs for all pathologies except pancreatitis, occasional failure to request imaging could be partly responsible for their low diagnostic accuracy.

**Fig. 4:**
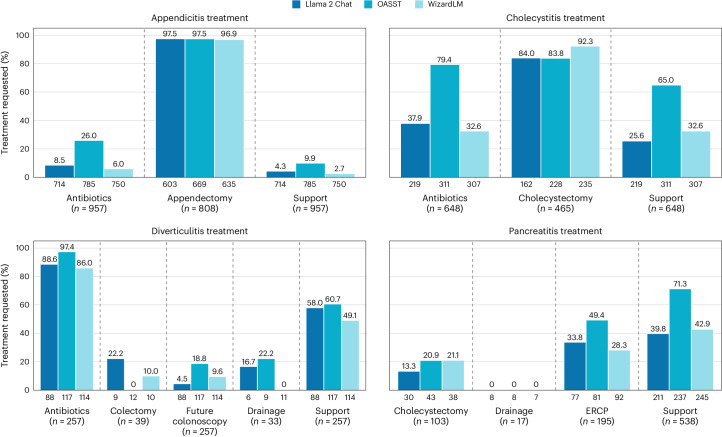
LLMs do not consistently recommend essential and patient-specific treatment. Expected treatments were determined based on clinical guidelines and actual treatments received by patients in the dataset. Models fail to recommend appropriate treatments especially for patients with more severe forms of the pathologies. We only scored models on the subset of patients that they correctly diagnosed and that actually received a specific treatment. For example, of the 957 patients with appendicitis, 808 received an appendectomy (indicated below the treatment name). Of those 808 patients, Llama 2 Chat correctly diagnosed 603 (indicated below the Llama 2 Chat bar). Of those 603 patients, Llama 2 Chat correctly recommended an appendectomy 97.5% of the time. ERCP, endoscopic retrograde cholangiopancreatography.

In addition to not following diagnostic guidelines, LLMs generally fail to adhere to treatment guidelines. We found that the LLMs consistently did not recommend appropriate and sufficient treatment, especially for patients with more severe forms of the pathologies (Fig. 4). While they are consistent in recommending some treatments such as appendectomy for appendicitis and antibiotics for diverticulitis, they rarely recommend other treatments when appropriate such as colectomy for patients with diverticulitis with perforated colons or drainage of infected pancreatic necrosis. Furthermore, they drastically undertreat appendicitis with regard to the necessity of antibiotics and providing support, undertreat diverticulitis with the need for a colonoscopy in the future to check for colon cancer, and undertreat pancreatitis with sufficient support. In summary, following the treatment recommendations of the models would negatively impact the health of patients, particularly those with more advanced stages of disease where indications for emergency operations were ignored.

Taken together, the lack of consistency of the LLMs in ordering all of the required tests for a diagnosis based on current guidelines indicates a tendency to diagnose before understanding or considering all the facts of the patient’s case. Such hasty decision-making combined with their poor diagnostic accuracy and treatment recommendations pose a serious risk to the health of patients without extensive clinician supervision and control.

### Current LLMs require extensive clinician supervision

In addition to consistently and safely arriving at the correct diagnosis and treatment plan, models must integrate into established clinical workflows to be useful. Central to this is the ability to follow instructions and generate answers so they can be easily processed and used by other parts of the clinic without physician supervision.

**Extended Data Fig. 6:**
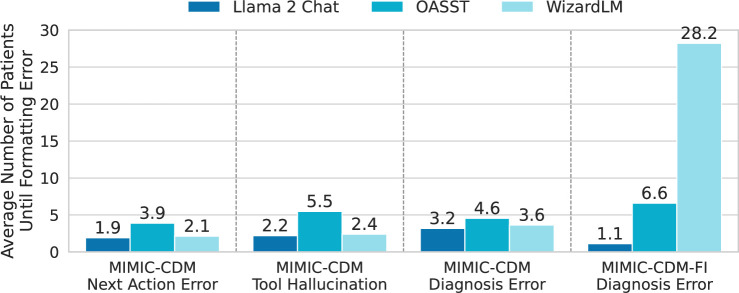
LLMs struggle to follow instructions. During the autonomous clinical decision making task, LLMs often introduce errors when providing the next action to take and hallucinate non-existent tools, up to once every two patients. Formatting errors while providing the diagnosis also regularly occur. In the clinic, extensive manual supervision would be required to ensure proper performance.

All models struggle to follow the provided instructions (Extended Data Fig. 6), making errors every two to four patients when providing actions and hallucinating nonexistent tools every two to five patients. When providing diagnoses on the MIMIC-CDM dataset, errors are made every three to five patients; while on the MIMIC-CDM-FI dataset, WizardLM is very consistent in following instructions, and Llama 2 Chat makes an error on almost every patient. While many of these errors are easily caught (Supplementary Section B), the error rate is so high that extensive manual controls would be necessary to ensure model output is being correctly interpreted, reducing their usefulness as autonomous clinical decision-makers.

**Extended Data Fig. 7:**
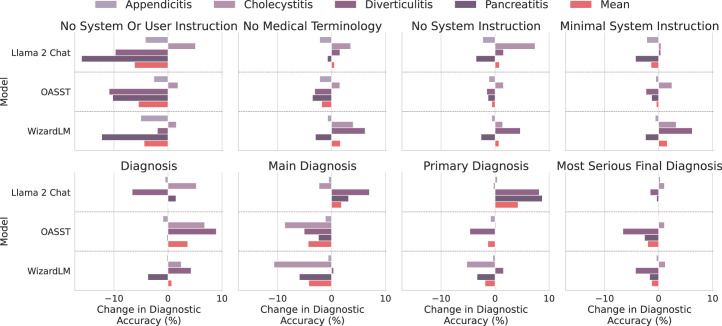
Small changes in instruction phrasing changes diagnostic accuracy. Often small changes in instructions, such as changing final diagnosis to main diagnosis or primary diagnosis, greatly affects the performance of the LLMs on the MIMIC-CDM-FI dataset. This would vary the quality of responses received depending on who is using the model.

Another key component that must be fulfilled before we consider integrating such models into real-world workflows is robustness. Models must not be sensitive to small changes in user instructions as their performance will then vary greatly based on who is interacting with them. On the MIMIC-CDM-FI dataset, we found that changes in instructions (Supplementary Section C) can lead to large changes (both positive and negative) in diagnostic accuracy (Extended Data Fig. 7). For example, large changes were seen when removing system and user instructions (up to +5.1% for Chat on cholecystitis, down to −16.0% for Chat on pancreatitis), or when removing all medical terminology from the system instruction (up to +6.2% for WizardLM on diverticulitis, down to −3.5% for OASST on pancreatitis). Additionally, we see that even minor changes in instructions can greatly change diagnostic accuracy such as asking for the ‘main diagnosis’ (up to +7.0% for Chat on diverticulitis, down to −10.6% for WizardLM on cholecystitis) or ‘primary diagnosis’ (up +8.7% for Chat on pancreatitis, down to −5.2% for WizardLM on cholecystitis) instead of ‘final diagnosis’. Models should be able to provide the most appropriate diagnosis given the situation, in this case the reason for the patient’s abdominal pain, and not be sensitive to minute changes in phrasing so as not to require extensive clinician training before use.

**Fig. 5:**
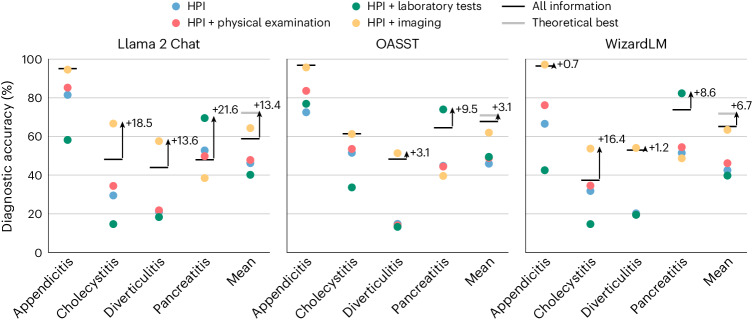
LLMs are sensitive to the quantity of information provided. We compared the performance of each model using all diagnostic information to using only a single diagnostic exam in addition to the history of present illness. For almost all diseases, providing all information does not lead to the best performance on the MIMIC-CDM-FI dataset. This suggests that LLMs cannot focus on the key facts and degrade in performance when too much information is provided. This poses a problem in the clinic where an abundance of information is typically gathered to holistically understand the patient’s health and being able to focus on key facts is an essential skill. The gray theoretical best line shows the mean performance if a clinician were to select the best diagnostic test for each pathology. HPI, history of present illness.

Furthermore, LLMs used for autonomous clinical decision-making should not degrade in performance when provided with relevant diagnostic information. We show that models perform worse when all diagnostic exams are provided, typically attaining their best performance when only a single exam is provided in addition to the history of present illness (Fig 5). Removing information greatly increases diagnostic accuracy, with cholecystitis diagnosis improving by 18.5% for the Chat and 16.5% for the WizardLM models when only providing radiologist reports, and pancreatitis diagnosis improving by 21.6% (Chat), 9.5% (OASST) and 8.6% (WizardLM) when only providing laboratory results. This reduces the usefulness of such models as they cannot simply be given all relevant information and be trusted to arrive at their best diagnosis. To optimize model performance, clinicians would have to decide which diagnosis is most likely to effectively filter the information presented, removing any benefit of deploying an autonomous clinical decision-making model.

**Fig. 6:**
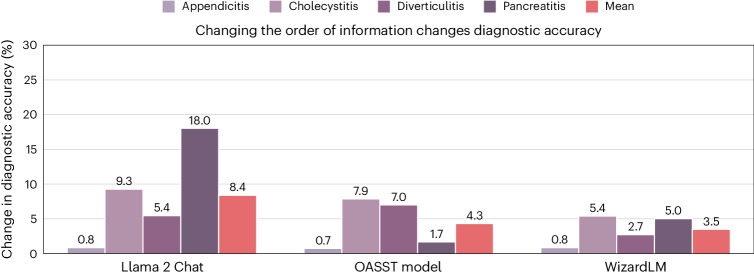
LLMs are sensitive to the order of information. By changing the order in which diagnostic information from MIMIC-CDM-FI is presented to LLMs, their diagnostic accuracy changes despite the information included staying the same. This places an unnecessary burden upon clinicians who would need to make preliminary diagnoses to decide the order in which they feed the models with information for best performance.

We further tested the diagnostic consistency of the models on the MIMIC-CDM-FI dataset by switching the order of the information from the canonical physical examination, then laboratory tests, then imaging, to all possible permutations thereof (history of present illness was always included first). We showed that all models have large ranges of performance, up to 18.0% (Chat—pancreatitis), 7.9% (OASST—cholecystitis) and 5.4% (WizardLM—cholecystitis; Fig. 6 and Supplementary Section D). Importantly, we found that the order of information that delivers the best performance for each model is different for each pathology (Supplementary Section E). This again reduces the benefits of deploying the models as clinicians must constantly consider and monitor in which order they provide the models with information, in a disease-specific manner, to not degrade performance.

In summary, extensive clinician supervision and prior evaluation of the most probable diagnosis would be required to ensure proper functioning of LLMs because they do not reliably follow instructions, perform better with a disease-dependent order of information and degrade in performance when given relevant information. Furthermore, their sensitivity to small changes in instructions that seem inconsequential to humans would require extensive clinician training to ensure good performance.

### First steps toward mitigating limitations of current LLMs

**Extended Data Fig. 8:**
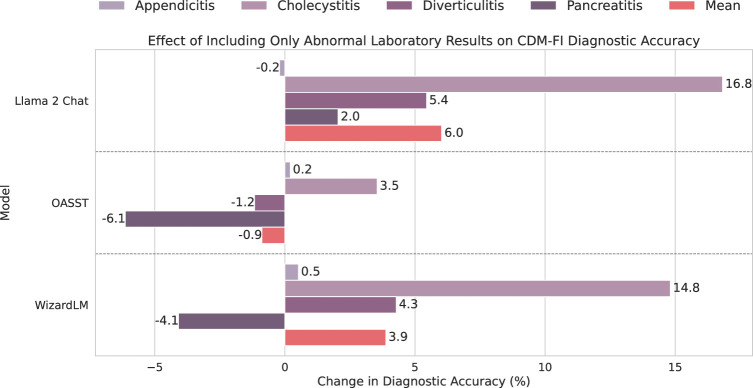
Filtering for only abnormal results generally improves LLM performance. Filtering to include only abnormal laboratory results using the laboratory reference ranges provided in MIMIC-IV database generally improves model performance, especially for the cholecystitis pathology. This allows the model to focus more on abnormal, pathological signals. While this approach is appropriate for models as they function today, healthy laboratory test results are an important source of information for clinicians and should not degrade model performance.

To help address some of the limitations found in this analysis, we explore simple modifications that can be done without retraining the model. One major limitation is that LLMs are currently limited in the amount of text they can read, which we address with an automatic summarization protocol (‘Evaluation framework’ in Methods). Removing such a summarization protocol resulted in marginal but consistent losses on the mean of −1.3% (Chat), −0.8% (OASST) and −0.5% (WizardLM), and particularly hurt the diagnosis of diverticulitis (−4.7%, Chat; −2.7%, OASST; −3.5%, Wizard; Supplementary Section F). Due to the inability of LLMs to reliably interpret laboratory results (Extended Data Fig. 4), even when provided with reference ranges, and their issues understanding larger quantities of information (Fig. 5), we found that filtering the laboratory results and removing all normal test results generally improved performance on the MIMIC-CDM-FI dataset (Extended Data Fig. 8). As many of our other analyses examine the general behavior of laboratory tests and their impact on model performance, we do not use this fix for any other sections of this work. While this filtering improves the performance of the LLMs as they function today, ideally a model would perform best with all available information.

## Discussion

The strong performance of LLMs on medical licensing exams has led to increased interest in using them in clinical decision-making scenarios involving real patients. However, medical licensing exams do not test the capabilities required for real-world clinical decision-making. We have evaluated leading open-access LLMs in an autonomous clinical decision-making scenario with thousands of real-world cases to assess their potential benefits and possible harms. By not only comparing their diagnostic performance against clinicians, but also testing their information-gathering abilities, adherence to guidelines and instruction-following capabilities as well as their robustness to changes in prompts, information order and information quantity, we move beyond simple evaluations of diagnostic accuracy and establish a range of characteristics that are necessary for safe and robust integration into the clinic. In this work, we have shown that current leading LLMs are unsuitable for autonomous clinical decision-making on all of these accounts.

The biggest barrier to using current LLMs either for autonomous clinical decision-making or as a second reader is that no model consistently reached the diagnostic accuracy of clinicians across all pathologies, with a further decrease in accuracy when having to gather diagnostic information themselves. To optimize performance on this specific clinical decision-making task, future studies could explore fine-tuning^40^ or prompt-tuning^41^ a base LLM as well as automated prompt engineering^42^. Another critical issue is that LLMs are unable to classify a lab result as normal or abnormal, even when provided with its reference range. This is underscored by the fact that presenting the model with only abnormal laboratory results generally improved diagnostic performance.

We further found that the models do not follow diagnostic guidelines, which is particularly problematic considering their low overall diagnostic accuracy, indicating a tendency to diagnose before fully understanding a patient’s case. Insufficient diagnostic information also negatively affected the treatment recommendations of LLMs, where we showed that models do not follow all established treatment guidelines, especially for severe cases. The hasty decision-making of the models combined with their low diagnostic performance and poor treatment recommendations pose a serious risk to the health of patients without extensive clinician supervision and control.

Beyond diagnostic accuracy, we extensively test models on their reliability and robustness, which are essential characteristics to ensure consistent and safe patient care. We found that models struggle to follow instructions, often hallucinating nonexistent tools and requiring continuous manual supervision to ensure proper performance. Models are also sensitive to seemingly inconsequential changes in instruction phrasing, requiring clinicians to carefully monitor the language they use to interact with the models to not degrade performance. Contrary to expectation, LLMs diagnose best when only a single diagnostic exam is provided rather than when given all relevant diagnostic information, demonstrating an inability to extract the most important diagnostic signal from the evidence. Future work could explore explicitly summarizing each new piece of evidence to further focus the model on only the most relevant information. Counterintuitively, we found models to be sensitive to the order in which information is presented, resulting in large changes in diagnostic accuracy despite identical diagnostic information. Importantly, all of these weaknesses are disease specific within each model, meaning that a different instruction, diagnostic test and order of tests achieved the best results for each pathology. Physicians would thus have to perform preliminary diagnostic evaluations in an attempt to maximize model performance according to their suspected diagnosis. This both increases the cognitive burden placed upon physicians and biases models toward the current preliminary diagnosis of the physician, removing the benefit of an unbiased second opinion.

Many of the current limitations of LLMs exposed in our study have been shown concurrently in domains outside medicine. It has been shown that LLMs are easily distracted^43^ and that their performance on tasks can vary by between 8% and 50% just by optimizing the instructions^44^. The sensitivity of LLMs to the order of presented information has been well documented on multiple-choice questions^45,46^ and information retrieval^47^. The difficulty LLMs have in interpreting numbers^48^ and solving simple arithmetic^49^ is an active research topic^50,51^. Even the largest models currently available, PaLM 2 and GPT-4, perform poorly on instruction-following tests^52^. Our analysis demonstrates how these current limitations of LLMs become harmful in medical contexts where robustness and consistency are essential.

We argue that these understudied aspects of model performance should become normal parts of medical model evaluations and that all of these issues must be addressed before LLMs can be considered for clinical decision-making.

While we have been able to demonstrate the limitations of current leading LLMs for clinical decision-making, we consider the following limitations of our study. First, as we are using a dataset of real-world clinical data, we must deny requests for data not in the dataset. However, as the MIMIC-IV database contains all data gathered during the hospital stay, we can assume that all information required for a diagnosis and treatment plan is contained within our dataset. Furthermore, being flexible enough to handle acute restrictions, such as unavailable imaging modalities or laboratory tests, and still come to a correct diagnosis is a desirable ability for any real-world clinical AI application. Due to this difficulty, we were lenient in our evaluation of the diagnoses, accepting alternative names for the pathologies, as long as they were medically correct (see Supplementary Section G). Additionally, both datasets and models have a clear bias toward the USA. The MIMIC data are in English and were gathered in an American hospital by doctors following American diagnostic and treatment guidelines. As the text used to train the LLMs is over 98% English and the most mentioned nationality by far is American (69.4%)^32^, the models are well suited for the constructed dataset, allowing us to use exclusively American guidelines for a fair evaluation. However, the generalizability of our results to other languages or countries with differing guidelines is unknown and needs to be explored in future work. To make sure the gains of advanced AI are equitably shared among all communities, there is a strong need for more clinical datasets in languages other than English and from countries other than the USA.

We believe that there exists great potential in using LLMs as clinical support systems with close collaboration between models and clinicians^21,27^; however, we have primarily evaluated the capabilities of models as autonomous decision-makers in this study. This allows for a fair and consistent evaluation of any current and future models, reducing the additional time required and costs generated by including multiple doctors in every evaluation. We welcome the use of our dataset and evaluation framework to test precisely such a collaborative effort between models and clinicians, where the summaries, actions and possible diagnoses of models returned throughout our framework are served to clinicians as an unbiased second opinion or to generate a list of possible diagnoses. Importantly, studies as to the impact of automation bias^53,54^ and human–AI interaction biases could also be explored in such a context^55–57^.

Lastly, by focusing on curating data for in-depth analysis of model behavior along every step of the diagnostic pathway, it was not feasible to include the full breadth of abdominal diseases. A fully autonomous clinical decision-making model must show strong performance across all possible pathologies of a particular initial complaint to guarantee adequate patient care; thus, it will be important to test future models on both additional diagnostic endpoints and a broader range of initial complaints.

In conclusion, our study presents an analysis of the capabilities of current state-of-the-art LLMs on real-world data in a realistic clinical decision-making scenario. Our main finding is that current models do not achieve satisfactory diagnostic accuracy, performing significantly worse than trained physicians, and do not follow treatment guidelines, thus posing a serious risk to the health of patients. This is exacerbated by the fact that they do not request the necessary exams for a safe differential diagnosis, as required by diagnostic guidelines, indicating a tendency to diagnose before fully understanding a patient’s case. Furthermore, we show LLMs are distracted by relevant diagnostic information, are sensitive to the order of diagnostic tests and struggle to follow instructions, prohibiting their autonomous deployment in the clinic and requiring extensive clinician supervision.

By sourcing our dataset from real clinical cases and closely aligning our evaluation criteria with official diagnostic and treatment guidelines, we present an analysis to help physicians understand how well LLMs would perform in the clinic today. While our findings cast doubt on the suitability of LLMs for clinical decision-making as they currently exist, we believe there lies great potential in their use after the issues raised are resolved.

By making our dataset and framework freely available, we hope to guide the development of the next generation of clinical AI models and contribute toward a future where physicians can feel confident in using safe and robust models to improve patient outcomes.

## Methods

### MIMIC-CDM dataset

We created our curated dataset of 2,400 patients, which we call MIMIC-CDM, using the MIMIC-IV Database^29^. The MIMIC-IV Database is a comprehensive, publicly available database managed by the Massachusetts Institute of Technology (MIT) of the de-identified electronic health records of almost 300,000 patients who presented to the Beth Israel Deaconess Medical Center in Boston, Massachusetts, USA from 2008 to 2019. It contains real patient cases from the hospital and includes all recorded measurements such as laboratory and microbiology test results, diagnoses, procedures, treatments and free-text clinical notes such as discharge summaries and radiologist reports.

In this work, we focus on four target pathologies for which we filter: appendicitis, cholecystitis, diverticulitis and pancreatitis. As we are only testing for these pathologies, we must ensure that they are the primary diagnosis and reason for the patient presenting to the emergency department and not merely a secondary diagnosis during a longer and more serious hospital admission. Thus, we first filtered patients for our targets using the diagnosis table, which contains all recorded diagnostic International Statistical Classification of Diseases and Related Health Problems (ICD) codes. Then, we manually checked the discharge diagnosis of each patient’s discharge summary and only included those patients whose very first primary diagnosis was one of our pathologies. If any other diagnosis was written in the discharge diagnosis before one of our targets, the patient’s case was removed from the dataset. If a patient was diagnosed with more than one of the four pathologies included in our analysis, the patient was removed from the dataset.

After filtering for the appropriate pathologies, we split the discharge summary into its individual sections, extracting the history of present illness and physical examination. First, we removed all patients who had pathology mentioned in their history of present illness as these admissions were mostly transfers where the diagnosis had already been established and the hospital admission data were thus missing the initial emergency department test results relevant for diagnostic purposes. Furthermore, we removed all patients who had no physical examination included as this is a crucial source of information according to the diagnostic guidelines of each pathology.

We gathered all laboratory and microbiology tests recorded during a patient’s hospital admission and those up to 1 day before admission. We included tests up to 1 day before admission as the MIMIC-IV documentation states that there are millions of laboratory tests that are not associated with any hospital admission but can be joined to patient stays using the patient’s ID, recorded test time and hospital admission time. The tests before the official start of the admission often had highly relevant values for diagnostic purposes and were thus included, although only if they were not associated with any other hospital admission. If a laboratory test was recorded multiple times, we included only the first entry in our dataset to simulate a therapy-naive diagnostic clinical decision-making scenario. Thus, we currently do not capture the changes in laboratory test values if the patient’s condition deteriorates over their hospital stay. This could be remedied by examining all time points and determining the most abnormal test result to be returned or by allowing multiple requests for laboratory tests to return successive test results. However, both of these approaches would widen the temporal gap of provided test results, possibly providing conflicting diagnostic signals. Considering LLMs also have poor temporal reasoning capabilities^69^, simply including the timestamp would most likely not be an adequate solution. Furthermore, we saved all reference ranges of the laboratory tests provided by MIMIC and established a comprehensive dictionary mapping possible synonyms and abbreviations of tests to their original entry to be able to interpret all requests of the LLMs for test results. This dictionary of synonyms was constantly expanded during initial testing of the models until no unrecognized tests were recorded. The dictionary also contains common laboratory test panel names that map to a list of the individual tests of that panel, such as complete blood count, basic metabolic panel, liver function panel, renal function panel and urinalysis, among others.

Similarly to the laboratory data, many radiology reports were not associated with any hospital admission, but their timestamp was a few hours before the recorded start of the hospital admission. These often contained diagnosis-relevant information and so we used the same 24-h inclusion criteria as those used for the laboratory results and again allowed only those exams not associated with any other hospital admission. Next, we established a list of uniquely identifying keywords for each anatomical region and imaging modality. We used this list of keywords to determine the region and modality of each included report from its MIMIC-IV provided exam name. Mappings were made for special exams such as CT urography to CT and MRCP to magnetic resonance imaging, to provide them if, for example, a CT scan or magnetic resonance imaging was requested, due to their low frequency. We also used this list when interpreting the model requests for imaging information during evaluation. We manually checked and adjusted the keywords until all reports in MIMIC-IV were correctly classified. Radiology reports were split into report sections and only the ‘findings’ section was included. This was done as many other sections such as ‘conclusions’ or ‘impressions’ contained the diagnosis of the radiologist, which would have made the task trivial.

Finally, all procedures and operations performed during a patient’s hospital stay were saved to understand which patient-specific treatments were undertaken. The procedures in the MIMIC-IV procedures table saved as ICD-9 and ICD-10 codes were extracted and combined with the free-text procedures section from each patient’s discharge summary. The free-text extraction from the discharge summary was required as many essential procedures, including surgeries, were often not included in the procedures table.

A final round of data cleaning replaced any remaining mentions of the primary diagnosis with three underscores ‘___’, which is used by MIMIC-IV to censor data such as a patient’s name or age. To increase data quality, we excluded patients who had no associated laboratory tests or for whom no abdominal imaging was recorded. The final dataset, MIMIC-CDM, contains 2,400 unique patients presenting to the emergency department with one of the four target pathologies (957 appendicitis, 648 cholecystitis, 257 diverticulitis, 538 pancreatitis) and whose makeup is detailed in Fig. 1a. The dataset contains physical examinations for all patients (2,400), 138,788 laboratory results from 480 unique laboratory tests and 4,403 microbiology results from 74 unique tests. Furthermore, MIMIC-CDM contains 5,959 radiology reports, including 1,836 abdominal CTs, 1,728 chest X-rays, 1,325 abdominal ultrasounds, 342 abdominal X-rays and 227 MRCP scans. Finally, there were 395 unique procedures recorded over all patients, with a total of 2,917 ICD procedures plus the 2,400 free-text procedures specified in the discharge summaries. Supplementary Section H shows the age, sex and race statistics of the patients in the dataset split up by pathology. As the reports provided were de-identified, the models did not have access to any of these characteristics during evaluation.

A second version of the dataset, which we call MIMIC-CDM-FI, combines all the information required for diagnosing each case and presents it all at once. Here we include the history of present illness, physical examination, all abdominal imaging and all laboratory data helpful for both reaching the correct diagnosis and ruling out differential diagnoses. To determine which laboratory data to include, we used the diagnostic guidelines of each disease: appendicitis^36^, cholecystitis^37^, diverticulitis^38^ and pancreatitis^39^. The specific tests included in each category can be found in Supplementary Section A. The information is presented in the order: history of present illness, physical examination, laboratory results, imaging. The imaging is ordered by chart time from earliest to latest.

### Reader study

For our reader study, we included two clinicians from the Klinikum Rechts der Isar Hospital of the Technical University of Munich, Germany (with 2 and 3 years of experience), one from the Ludwig Maximilian University Hospital in Munich, Germany (4 years of experience) and one from the Christiana Care hospital in Delaware, USA (29 years of experience).

All four of the hospitalists are internal medicine physicians with emergency department experience.

A subset of 80 representative patients of the MIMIC-CDM-FI dataset was randomly selected to be used for comparison with the physicians. The subset was evenly split between the four target pathologies with 20 patients randomly selected from each pathology and matching the makeup of the full dataset (Supplementary Section H). To mitigate the risk of physicians recognizing the pattern of four distinct target pathologies, a further five patients presenting with gastritis, a urinary tract infection, esophageal reflux and a hernia were included. For the physicians, the data were prepared as a PDF and the information was provided exactly in the same order and quantity as for the models. Reference ranges were included when provided by MIMIC-IV. The abbreviations in the history of present illness and physical examination were replaced with unabbreviated text for the German doctors, as they were unfamiliar with US-specific abbreviations. The models performed worse with unabbreviated text (Supplementary Section I). The laboratory data were provided as a table in the PDF to increase readability. Thus, the final dataset used in the reader study spanned 100 patients, of whom 80 are part of MIMIC-CDM-FI. Each hospitalist was instructed to provide the primary pathology affecting the patient and was given the same 100 patients in a random order to diagnose.

Each LLM model was evaluated 20 times, using different random seeds, over the subset of 80 patients to increase statistical power. All statistical tests were corrected for multiple comparisons (‘Metrics and statistical analysis’ in Methods).

Our comparison between models and clinicians included only three doctors in residency from Germany and one senior hospitalist from the United States. Increasing the diversity and number of clinicians as well as the number of patient cases evaluated would give a more nuanced view of model performance compared to practicing hospitalists. Future models could possibly soon reach or even outperform clinicians in residency and thus provide a low-cost, interactive second opinion to consult, as is already the case for AI models in other areas such as mammography screening^70^.

### Evaluation framework

To realistically test the capabilities of LLMs on the task of clinical decision-making, we simulated a clinical environment in which a patient presents to the emergency department with acute abdominal pain and information must be iteratively gathered before a final diagnosis is made. The LLM is tasked with the Clinical Decision Making (CDM) task (Supplementary Section C.1), which instructs it to consider a patient’s symptoms and gather information to come to a diagnosis and treatment plan while also explaining the two formats it should answer with. Both formats ask the LLM to ‘think’ (that is, consider the evidence, which has been shown to improve the quality of reasoning and future actions^71,72^), and then either request more information or provide a diagnosis and treatment plan. This allows the model to summarize the most important information into the thoughts section, which guides their choice of action or diagnosis. If it chooses to request more information, it must state ‘action’ followed by either ‘physical examination’, ‘laboratory test’ or ‘imaging’. Additionally, it must provide an ‘action input’, which specifies what information is desired from the action (that is, ‘complete blood count’ or ‘abdominal ultrasound’). The ‘action input’ field is ignored if a physical examination is requested. The second format is to be used when the model decides enough information has been gathered for a diagnosis, and asks the model to consider the evidence one last time and then provide a final diagnosis and treatment plan.

The model is initially presented with these instructions and the history of present illness of the patient and then prompted to record its ‘thoughts’; thus, beginning the clinical decision-making task. Outputs are generated until either a stop token is reached or the model generates the ‘observation(s)’ phrase, indicating that it has reached the end of its action request and would potentially start hallucinating the result of its request. We stop model text generation here and then examine the response of the model, extracting which action was desired and what the input to that action is. If the model does not follow the instructions and, for example, writes ‘perform a physical examination’ instead of ‘action: physical examination’, we still provide the appropriate information but record every instance of it not following instructions for our evaluations (Extended Data Fig. 6). We call these errors ‘next action errors’.

If the requested information is available for that patient case, we return it and prompt the model again to consider the evidence. If the information is not available, we inform the model and ask for an alternative action. We return only the laboratory tests and radiologist reports that were specifically requested. Laboratory tests are compared to the previously mentioned dictionary of available tests to return the best match. If no match is found, ‘NA’ is returned. Requests for imaging have the exam modality and anatomical region extracted using the aforementioned keyword lists and used to match against those saved for that patient in MIMIC-CDM. If multiple reports exist for a modality and region combination, we return the first report chronologically. The next request for an imaging examination of that modality and region will return the next report chronologically. Once there are no reports left to return, we inform the model that we can no longer provide reports of that modality and region combination. If a physical examination was requested, we return the entire physical examination regardless of any specifications made in the ‘action input’ field. We do this because we consider it best practice to perform a complete physical examination of a patient rather than only partially, and reliably separating a physical examination report into its parts is difficult due to their free-form and heavily abbreviated style. If an invalid tool is requested (‘hallucinated’), we state that the tool does not exist and remind it which tools are available, or that it should make a diagnosis and provide a treatment plan. These occasions are also recorded as tool hallucinations for our evaluations (Extended Data Fig. 6). An example exchange between an LLM and our framework can be seen in Fig. 1b and Supplementary Section J. We repeat this process, prompting the LLM to think and in turn receiving requests for information. Once the model decides that it has gathered sufficient information, it outputs its final diagnosis and treatment plan, ending the clinical decision-making task for that patient. The final diagnosis is then evaluated to see if it contains the recorded pathology of the patient. In addition to a direct match of the pathology name (that is, appendicitis, cholecystitis, diverticulitis or pancreatitis), we allow for a range of alternative phrasings as long as they are medically correct (Supplementary Section G). If multiple diagnoses are given, we only examine the first diagnosis mentioned. This is how we calculate the diagnostic accuracy for all analyses. A new instance of the task is then started for the next patient.

As LLMs can only take a limited amount of words as input, with all models tested in this study having a limit of 4,096 tokens or approximately 2,400 words, we monitor the length of the conversation. If we approach the input limit of the model, we ask it to summarize the information it has received so far to reduce the length of the conversation (Supplementary Section C.2). We first summarize each gathered piece of information individually, leaving the initial history of present illness and instructions untouched. As LLMs have no memory and interpret each request independently, we replace the original pieces of information with the summaries. If we have summarized all steps of the interaction and still approach the limit of the model, we force the generation of a diagnosis and treatment plan.

For the MIMIC-CDM-FI dataset, we instruct the model to consider the facts of the case and then provide a diagnosis and only a diagnosis (Supplementary Section C.3). As previously explained, the MIMIC-CDM-FI dataset includes the history of present illness, physical examination, all relevant laboratory results and every radiologist report where the abdominal region was inspected. Radiologist reports of other regions were not included due to the input length limits of the models. If including all of this information exceeds the input length of the model, we ask the LLM to summarize each radiologist report individually. If the input length is still exceeded, we ask the LLM to summarize all imaging information at once. In the rare cases where the input length continues to be exceeded, we remove words from the final imaging summary until there is enough space for a diagnosis (that is, 25 tokens or 20 words).

### Analysis

#### Treatment requested

To evaluate the ability of the LLMs to recommend appropriate treatments, we used the aforementioned guidelines to extract the possible treatments for each pathology and then to classify each treatment as either essential (for example, antibiotics and support) or case specific (for example, appendectomy, cholecystectomy and drainage). For each patient, we then determined if the case-specific treatment was appropriate by matching against the actual operations performed on that patient, read from the MIMIC-CDM dataset. We evaluated a model’s treatment recommendation only when it correctly diagnosed a patient since an inaccurate diagnosis likely leads to inappropriate treatment. For support, we expect mentions of fluids, pain management or monitoring for appendicitis, cholecystitis and diverticulitis. As it is the main form of treatment for pancreatitis, we expect mentions of all three for this disease.

#### Instruction-following capabilities

During the clinical decision-making process, we provide clear instructions to the models as to how they should provide their requests and diagnosis, as well as which tools are available to them (Supplementary Section C.1). For example, diagnostic tools must be written in the ‘action’ field and desired tests must be specified in the ‘action input’ field, and not in the middle of a paragraph surrounded by other text. This is essential to ensure that the desired tests can be consistently extracted so no manual clinician supervision and interpretation is required. Through extensive comparisons of LLM outputs with dictionaries of known exams and their synonyms, we go to great lengths to understand what tests are requested, even if the models do not follow our schema, recording every time they fail to follow instructions. We investigated the capabilities of models to follow our instructions at three time points during our analysis: (1) when providing the next action to take, (2) when requesting a tool and (3) when providing a diagnosis.

### Models

#### Model selection

An overview of the models included and considered is given in Table 1.

**Table 1:** An overview of the considered LLMs and their properties

Model	Base	Parameters	Training dataset	Downloadable
Llama 2 Chat^32^	Llama 2 (ref. ^32^)	70B	Public data^a^	*✓*
OASST^33^	Llama 2 (ref. ^32^)	70B	Public data^a^, https://huggingface.co/OpenAssistant/llama2-70b-oasst-sft-v10/, open-source data	*✓*
WizardLM^34^	Llama 2 (ref. ^32^)	70B	Public data^a^, Evol-Instruct generated^34^	*✓*
Clinical Camel^19^	Llama 2 (ref. ^32^)	70B	Public data^a^, https://sharegpt.com/; ShareGPT; PubMed articles (before 2021)^19^, MedQA^13^	*✓*
Meditron^35^	Llama 2 (ref. ^32^)	70B	Public data^a^, https://huggingface.co/datasets/epfl-llm/guidelines/; clinical guidelines, public PubMed abstracts^35^, public PubMed papers^35^, RedPajama^58^	*✓*
Chat-GPT^59^	GPT3.5 (ref. ^60^)	???	User conversations^b^, Common Crawl^61^, WebText2 (ref. ^62^), Books1 (ref. ^63^), Books2 (ref. ^63^), Wikipedia	✗
GPT-4 (ref. ^64^)	???	???	???	✗
Med-PaLM^9^	Flan-PaLM^65^	540B	Webpages^b^, Wikipedia^b^, social media^b^, GitHub^b^, news articles^b^, books^b^, 473 instruction fine-tuning datasets^65^, HealthSearchQA^9^, MedicationQA^66^, LiveQA^67^	✗
Med-PaLM 2 (ref. ^8^)	PaLM 2 (ref. ^68^)	340B	Web Documents^b^, books^b^, code^b^, mathematics^b^, conversational data^b^, MedQA^13^, HealthSearchQA^9^, MedicationQA^66^, LiveQA^67^	✗

Due to the data usage agreement of MIMIC-IV, only open-access models that can be downloaded can be used with the data; thus, only LLMs based on Llama 2 were used in this study. ??? indicates no information has been made public.

^a^Meta defines ‘public data’ as a ‘mix of data from publicly available sources’.

^b^No further information provided.

When deciding which models to test, we started by only considering models with a context length of 4,096 tokens due to the large amounts of text contained within the MIMIC-CDM clinical cases. The context length defines how many combined tokens an LLM can read and write. For example, if a model has a context length of 2,048 and receives an input of 2,000 tokens, it can only generate 48 new tokens. A minimum length of 4,096 is required, as the average number of tokens of relevant information per patient case in MIMIC-CDM-FI is 2,080 tokens with a maximum count of 15,023 tokens. If one considers the extra tokens that are required for the back-and-forth information gathering using MIMIC-CDM data, this quickly exceeds the limit of 2,048 tokens of smaller models (context length windows almost always differ in powers of 2).

Next, we considered which open-access models performed best on medical reasoning tasks. To gauge model strength, we used the MedQA^13^ dataset as it comprises 12,723 questions from the USMLE and is thus a good gauge of general medical knowledge contained within the model. At the time of writing, Llama 2 is the leading open-access model on the MedQA (USMLE) dataset, with the 70B model achieving a score of 58.4 (ref. ^35^), exceeding that of GPT3.5, which scored only 53.6 (ref. ^10^).

To effectively complete the clinical decision-making task without specific fine-tuning to the task and format, the model must be fine-tuned to instructions. Instruction fine-tuning involves training a model to adapt to a wide range of new tasks so that it can, with minimal instruction or example, complete an unseen task, like our clinical decision-making objective. The most popular and performant instruction fine-tuned versions of Llama 2 are Llama 2 Chat, fine-tuned by Meta themselves; WizardLM, fine-tuned by Microsoft using evolutionary algorithm (Evo-Instruct)-generated training data; and OASST, fine-tuned using a crowd-sourced collection of 161,443 messages. Currently the only two existing medically fine-tuned versions of Llama 2 with a context length of 4,096 and 70B parameters are Clinical Camel and Meditron, respectively. Neither has been extensively fine-tuned to instructions and thus they both generated nonsensical and repetitive responses to the clinical decision-making objective using MIMIC-CDM data.

Currently, the most popular and leading closed-source LLMs for medical question answering are Chat-GPT (MedQA: ~53.6)^10^, GPT-4 (MedQA: 90.2)^20^, Med-PaLM (MedQA: 67.2)^8^ and Med-PaLM 2 (MedQA: 86.5)^8^. As previously stated, due to the signed data usage agreements of the MIMIC-IV database, the data cannot be sent to external servers^73^, precluding its use with closed-source models that are only accessible through an API and whose models cannot be downloaded.

Furthermore, Chat-GPT is fine-tuned primarily through user conversations with the model, and since it is impossible to know if portions of the MIMIC-IV database have already been used for queries by users less aware of the data usage agreement^73^, the data could already have been seen by the model during training, invalidating any results it produces. Little to no information is known about the training data of GPT-4, giving rise to analogous concerns about its performance. While the exact pretraining data of Llama 2 are also not known, Meta has stated that it only used ‘publicly available online data’, which strongly mitigates the risk of MIMIC-IV data having been used. Med-PaLM and Med-PaLM 2 achieve strong scores on MedQA but the exact data used for training are unknown, the models are only accessible through an API, and access to the models is currently unavailable for all researchers. Repeated requests for access were denied.

We strongly agree with the current sentiment that open-source models must drive progress in the field of medical AI due to patient privacy and safety concerns, corporate lack of transparency and the danger of unreliable external providers^74^. It is a serious risk to patient safety if key medical infrastructure is based on external company APIs and models whose performance could change erratically with updates and which could generally be deactivated for any reason.

For each model tested, we downloaded and used the GPTQ quantized version from Hugging Face, the central repository for all LLM models. GPTQ quantization reduces the numerical precision of the weights while monitoring the generated output to reduce the GPU memory requirements of a model while preserving performance^75^. The GPTQ parameters of the downloaded models were: 4 bits, 32 group size, act order true, 0.1 damp% and 4,096 sequence length. This gives the highest possible inference quality while reducing model size to around 40 GB, which can fit onto a single A40 GPU. This reflects an economically realistic scenario of a single high-end GPU being used to host the model to run the clinical decision-making task. A fixed seed of 2023 and greedy decoding were used for all experiments making all results deterministic and reproducible, except for the evaluation on the subset of 80 patients for comparison with clinicians where 20 different seeds were used for increased statistical power.

#### An overview of model strengths and weaknesses

Among the models tested in this study, we found that OASST performed best overall as it had decent diagnostic accuracy, generally requested appropriate laboratory exams and was most robust to changes in information quantity. Llama 2 Chat had the worst overall diagnostic accuracy, often refused to follow instructions and was heavily influenced by the order and quantity of information, but it was the only model to consistently ask for a physical examination. WizardLM was the most robust to changes in the order of diagnostic exams and followed instructions well when returning diagnoses, but was the worst at following diagnostic guidelines, failing to consistently order physical examinations and necessary laboratory tests. Despite the performance of OASST being generally better than Chat and WizardLM across the diverse set of analyses included in this study, it is still not currently suitable for clinical use due to its inferior performance compared to clinicians, broad failure to order correct treatments and general lack of robustness. While one of the medical-domain models, Clinical Camel, achieved the highest diagnostic accuracy (mean = 73% versus OASST mean = 68%; Extended Data Fig. 1), its inability to participate in the iterative clinical decision-making task precluded it from evaluations of its robustness and consistency, which we believe to be essential to ensure safe deployment in the clinic. Other LLMs such as Chat-GPT, GPT-4, Med-PaLM and Med-PaLM 2 could not be tested due to the data privacy and usage agreements of MIMIC-IV, highlighting the risk of using corporate models in a sensitive area such as medicine, where patient privacy, transparency and reliability are essential^74,76^.

### Metrics and statistical analysis

By focusing on curating data for in-depth analysis of model behavior along every step of the diagnostic pathway, it was not feasible to include the full breadth of abdominal diseases. However, for an accurate counting of false positives and true negatives, the patients in our dataset and patients presenting to a hospital with abdominal pain should have a similar diversity of disease. As this is not the case, metrics based on the false positives and true negatives could potentially be misleading.

Given these constraints, we calculate and use the per-class accuracy throughout our work, which is the only metric that can be calculated without bias as it only requires the samples of a single class. For each model and disease combination, we divided the number of correct diagnoses by the total number of patients with that disease. Additional metrics are included in Supplementary Section K, although they are to be interpreted with caution.

All statistical tests were conducted using the Python programming language (version 3.10) and the SciPy library. Comparisons of means were tested for statistical significance using two-sided Student’s *t*-tests with unequal variances (tested through Bartlett’s tests). To account for multiple comparisons, *P* values were Bonferroni corrected with a multiplier of 5 for the comparison of the doctors against the models and 3 for the comparison of the specialist and generalist models.

### Reporting summary

Further information on research design is available in the Nature Portfolio Reporting Summary linked to this article.

## Online content

Any methods, additional references, Nature Portfolio reporting summaries, source data, extended data, supplementary information, acknowledgements, peer review information; details of author contributions and competing interests; and statements of data and code availability are available at 10.1038/s41591-024-03097-1.

## Supplementary information

Supplementary Information Supplementary Sections A–N (contain information on the evaluation framework and aspects of model evaluation, model performance, supplementary discussion and dataset statistics) and Tables 1–9.

Reporting Summary

## Data Availability

The dataset is available to all researchers who create an account on https://physionet.org/ and follow the steps to gain access to the MIMIC-IV database (https://physionet.org/content/mimiciv/2.2/). Access is given after completing the ‘CITI data or specimens only research’ training course. The data use agreement of PhysioNet for ‘credentialed health data’ must also be signed. The dataset can then be recreated using the code found at https://github.com/paulhager/MIMIC-Clinical-Decision-Making-Dataset/. The generated dataset can also be directly downloaded from PhysioNet oncec (see above) via https://www.physionet.org/content/mimic-iv-ext-cdm/1.0/.

## Code availability

The evaluation framework used for this study can be found at https://github.com/paulhager/MIMIC-Clinical-Decision-Making-Framework/. The analysis framework to evaluate all results, generate all plots and perform all statistical analysis can be found at https://github.com/paulhager/MIMIC-Clinical-Decision-Making-Analysis/. All code uses Python v3.10, pytorch v2.1.1, transformers v4.35.2, spacy v3.4.4, langchain v0.0.339, optimum v1.14, thefuzz v0.20, exllamav2 v0.0.8, nltk v3.8.1, negspacy v1.0.4 and scispacy v0.5.2. The code to create the dataset uses Python v3.10 and pandas v2.1.3.
