# Supplementary material for: Evaluation and mitigation of the limitations of large language models in clinical decision-making
Source: Nat Med. 2024 Jul 4;30(9):2613–22. doi: 10.1038/s41591-024-03097-1 (PMC11405275; doi:10.1038/s41591-024-03097-1)
Supplement: Supplementary file 1 — Supplementary Sections A–N (contain information on the evaluation framework and aspects of model evaluation, model performance, supplementary discussion and dataset statistics) and Tables 1–9. [file 41591_2024_3097_MOESM1_ESM.pdf]

nature medicine

Supplementary information https://doi.org/10.1038/s41591-024-03097-1

Evaluation and mitigation of the limitations of large language models in clinical decision-making

In the format provided by the authors and unedited

A Laboratory Test Categories

**Pathologies**	**Category**	**Items**
Appendicitis<br>Cholecystitis<br>Diverticulitis<br>Pancreatitis	Inflammation	White Blood Cells<br>WBC Count<br>C-Reactive Protein<br>High-Sensitivity CRP
Cholecystitis	Gallbladder	Bilirubin<br>Gamma Glutamyltransferase
Cholecystitis	Liver	Alanine Aminotransferase (ALT)<br>Asparate Aminotransferase (AST)
Pancreatitis	Pancreas	Amylase<br>Lipase
Pancreatitis	Pancreatitis Severity	Hematocrit<br>Urea Nitrogen<br>Triglycerides<br>Calcium<br>Potassium

Table 1: **Laboratory test categories and their individual items.** Listed here are the individual laboratory test category items defined using the guidelines for appendicitis[17], cholecystitis[47], diverticulitis [26], and pancreatitis[35]. At least one of these tests must have been ordered either individually or as part of a panel for the category to be counted.

B LLMs Struggle to Follow Instructions

Error Name	Error Example	Corrected Example
Next Action Error	Order labs	Action: Laboratory Tests
	Perform a physical examination	Action: Physical Examination
	Run a CT scan of the abdomen	Action: Imaging<br>Action Input: Abdominal CT
Tool Hallucination	Action: Consult specialist	
	Action: Consider the evidence	
Diagnosis Error	Final Diagnosis: Based on the patient's symptoms, laboratory results, and imaging studies, the final diagnosis is: Appendicitis	Final Diagnosis: Appendicitis
	Final Diagnosis: Acute Gallstone Pancreatitis. The patient's symptoms of abdominal pain, nausea, vomiting, and fever, along with the laboratory results of elevated serum amylase and lipase levels, suggest a diagnosis of acute pancreatitis.	Final Diagnosis: Acute Gallstone Pancreatitis
	Final Diagnosis: Appendicitis, abdominal pain, and elevated white blood cells	Final Diagnosis: Appendicitis

Table 2: **Example errors.** Examples of the types of errors commonly made by models when providing actions and diagnoses. The corrected example in the desired format is also provided. Note that tool hallucination examples are simply not valid actions and so there are no corrected examples provided.

C Prompts

C.1 CDM Template

```
{system_tag_start}You are a medical artificial intelligence assistant. You give helpful,
detailed and factually correct answers to the doctors questions to help him in his
clinical duties. Your goal is to correctly diagnose the patient and provide treatment
advice. You will consider information about a patient and provide a final diagnosis.

You can only respond with a single complete

Thought:
Action:
Action Input:

format OR a single

Thought:
Final Diagnosis:
Treatment:

format. Keep all reasoning in the Thought section. The Action, Action Input, Final
Diagnosis, and Treatment sections should be direct and to the point. The results of
the action will be returned directly after the Action Input field in the "Observation:"
field.

Format 1:

Thought: (reflect on your progress and decide what to do next)
Action: (the action name, should be one of [{tool_names}])
Action Input: (the input string to the action)
Observation: (the observation from the action will be returned here)

OR

Format 2:

Thought: (reflect on the gathered information and explain the reasoning for the final
diagnosis)
Final Diagnosis: (the final diagnosis to the original case)
Treatment: (the treatment for the given diagnosis)

The tools you can use are:

Physical Examination: Perform physical examination of patient and receive the
observations.
Laboratory Tests: Run specific laboratory tests and receive their values. The specific
tests must be specified in the 'Action Input' field.
Imaging: Do specific imaging scans and receive the radiologist report.
Scan region AND modality must be specified in the 'Action Input'
field.{add_tool_descr}{system_tag_end}{user_tag_start}{examples}Consider the following
case and come to a final diagnosis and treatment by thinking, planning, and using the
aforementioned tools and format.

Patient History:
{input}{user_tag_end}{ai_tag_start}Thought:{agent_scratchpad}
```

C.2 CDM Observation Summarize Template

```
{system_tag_start}You are a medical artificial intelligence assistant. Your goal is to
effectively, efficiently and accurately reduce text without inventing information. You
want to return verbatim observations that are abnormal and of interest to a possible
diagnosis of the patient. Normal observations can be combined. Do not invent information.
Use medical abbreviations when possible to save characters. Put the most important
information first.{system_tag_end}{user_tag_start}Please summarize the following result:
{observation}{user_tag_end}{ai_tag_start}
Summary:
```

C.3 CDM-FI Template

```
{system_tag_start}You are a medical artificial intelligence assistant. You
directly diagnose patients based on the provided information to assist a doctor
in his clinical duties. Your goal is to correctly diagnose the patient. Based
on the provided information you will provide a final diagnosis of the most
severe pathology. Don't write any further information. Give only a single
diagnosis.{system_tag_end}{fewshot_examples}{user_tag_start}Provide the most likely final
diagnosis of the following patient.

{input}{diagnostic_criteria}{user_tag_end}{ai_tag_start}Final Diagnosis:
```

C.4 Reference Range Test Zeroshot Template

```
{system_tag_start}You are a technical AI assistant working in a laboratory that handles
tests for a hospital. You are good at interpreting numbers. You are responsible for
reviewing the results of lab tests and determining whether they are Low, Normal,
or High. You will be given the test, its value and then the reference range for
that test, which will be written as "Reference Range [Lower Reference Range -
Upper Reference Range]". You will write just one word, indicating if the test
results are Low, Normal, or High. Do not write anything other than your one word
answer.{system_tag_end}{user_tag_start}{lab_test_string_rr}{user_tag_end}{ai_tag_start}Test
Result:
```

C.5 CDM-FI No Final Template

```
{system_tag_start}You are a medical artificial intelligence assistant. You directly
diagnose patients based on the provided information to assist a doctor in his clinical
duties. Your goal is to correctly diagnose the patient. Based on the provided information
you will provide the diagnosis. Don't write any further information. Give only a single
diagnosis.{system_tag_end}{fewshot_examples}{user_tag_start}Provide the diagnosis of the
following patient.

{input}{diagnostic_criteria}{user_tag_end}{ai_tag_start}Diagnosis:"""
```

C.6 CDM-FI Main Diagnosis Template

```
{system_tag_start}You are a medical artificial intelligence assistant. You directly
diagnose patients based on the provided information to assist a doctor in his clinical
duties. Your goal is to correctly diagnose the patient. Based on the provided information
you will provide the main diagnosis. Don't write any further information. Give only a
single diagnosis.{system_tag_end}{fewshot_examples}{user_tag_start}Provide the main
diagnosis of the following patient.

{input}{diagnostic_criteria}{user_tag_end}{ai_tag_start}Main Diagnosis:"""
```

C.7 CDM-FI Primary Diagnosis Template

```
{system_tag_start}You are a medical artificial intelligence assistant. You directly
diagnose patients based on the provided information to assist a doctor in his clinical
duties. Your goal is to correctly diagnose the patient. Based on the provided information
you will provide the primary diagnosis. Don't write any further information. Give only
a single diagnosis.{system_tag_end}{fewshot_examples}{user_tag_start}Provide the primary
diagnosis of the following patient.

{input}{diagnostic_criteria}{user_tag_end}{ai_tag_start}Primary Diagnosis:"""
```

C.8 CDM-FI No System Template

```
{system_tag_start}{system_tag_end}{fewshot_examples}{user_tag_start}Provide the most
likely final diagnosis of the following patient.

{input}{diagnostic_criteria}{user_tag_end}{ai_tag_start}Final Diagnosis:"""
```

C.9 CDM-FI No User Template

```
{system_tag_start}You are a medical artificial intelligence assistant. You
directly diagnose patients based on the provided information to assist a doctor
in his clinical duties. Your goal is to correctly diagnose the patient. Based on
the provided information you will provide a final diagnosis of the most severe
pathology. Don't write any further information. Give only a single diagnosis.
{system_tag_end}{fewshot_examples}{user_tag_start}{input}{diagnostic_criteria}
{user_tag_end}{ai_tag_start}Final Diagnosis:"""
```

C.10 CDM-FI No Medical Template

```
{system_tag_start}You are an artificial intelligence assistant. You answer questions to
the best of your abilities. Think hard about the following problem and then provide an
answer.{system_tag_end}{fewshot_examples}{user_tag_start}Provide the most likely final
diagnosis of the following patient.

{input}{diagnostic_criteria}{user_tag_end}{ai_tag_start}Final Diagnosis:"""
```

C.11 CDM-FI Serious Final Template

```
{system_tag_start}You are a medical artificial intelligence assistant. You
directly diagnose patients based on the provided information to assist a doctor
in his clinical duties. Your goal is to correctly diagnose the patient. Based
on the provided information you will provide a final diagnosis of the most
severe pathology. Don't write any further information. Give only a single
diagnosis.{system_tag_end}{fewshot_examples}{user_tag_start}Provide the most serious
final diagnosis of the following patient.

{input}{diagnostic_criteria}{user_tag_end}{ai_tag_start}Final Diagnosis:"""
```

C.12 CDM-FI Minimal System Template

```
{system_tag_start}You are a medical artificial intelligence assistant. You diagnose
patients based on the provided information to assist a doctor in his clinical
```

```
duties.{system_tag_end}{fewshot_examples}{user_tag_start}Provide the most likely final
diagnosis of the following patient.

{input}{diagnostic_criteria}{user_tag_end}{ai_tag_start}Final Diagnosis:"""
```

C.13 CDM-FI No System No User Template

```
{system_tag_start}{system_tag_end}{fewshot_examples}{user_tag_start}{input}
{diagnostic_criteria}{user_tag_end}{ai_tag_start}Final Diagnosis:"""
```

C.14 CDM-FI No Diagnosis Prompt Template

```
{system_tag_start}You are a medical artificial intelligence assistant. You
directly diagnose patients based on the provided information to assist a doctor
in his clinical duties. Your goal is to correctly diagnose the patient. Based
on the provided information you will provide a final diagnosis of the most
severe pathology. Don't write any further information. Give only a single
diagnosis.{system_tag_end}{fewshot_examples}{user_tag_start}Provide the most likely final
diagnosis of the following patient.

{input}{diagnostic_criteria}{user_tag_end}{ai_tag_start}"""
```

D LLMs are Sensitive to the Order of Information

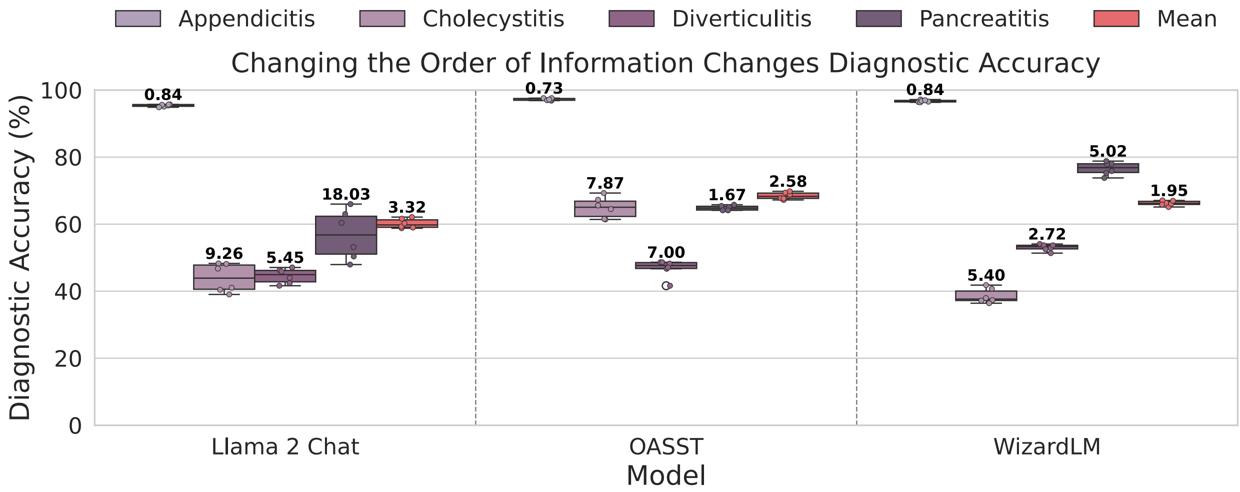

Figure 1: **Boxplots showing LLMs are sensitive to the order of information.** By mixing the order in which information is presented to LLMs, their diagnostic accuracy changes despite the information included staying the same. This places an unnecessary burden upon clinicians who would need to consider and monitor the order in which they feed the models with information. The boxplots are calculated using the possible permutations of ordering physical examinations, laboratory results and imaging results which are shown as colored dots (n=6). The center line marks the median of the data. The edges of the box are the lower and upper bound demarking 25% and 75% of the data points. The whiskers show the minimum and maximum values that are a maximum of 1.5 times the interquartile range from the edges of the box. Outliers are shown separately. The value above each top-whisker shows the difference between the best performing and worst performing order.

E LLMs are sensitive to information order

Experiment<br>Pathology	Imaging, Lab<br>Physical	Imaging, Physical<br>Lab	Lab, Imaging<br>Physical	Lab, Physical<br>Imaging	Physical, Imaging<br>Lab	Physical, Lab<br>Imaging
Appendicitis	**95.72**	**95.72**	95.61	94.88	95.40	94.98
Cholecystitis	39.04	40.43	46.76	48.30	41.05	**64.35**
Diverticulitis	45.91	46.30	41.63	42.41	47.08	**49.03**
Pancreatitis	60.41	**65.99**	53.16	50.37	63.01	48.14
Mean	60.27	62.11	59.29	58.99	61.64	**64.13**

Table 3: **Llama 2 Chat diagnostic performance with different diagnostic information orderings.** Changing the order of the presented information changes diagnostic accuracy. Crucially, the best order (bold) is disease specific, meaning a clinician must deliver a preliminary diagnosis to ensure proper model performance, eliminating many of the benefits of an AI clinical decision making system.

Experiment<br>Pathology	Imaging, Lab<br>Physical	Imaging, Physical<br>Lab	Lab, Imaging<br>Physical	Lab, Physical<br>Imaging	Physical, Imaging<br>Lab	Physical, Lab<br>Imaging
Appendicitis	97.18	**97.60**	96.97	96.97	**97.60**	97.39
Cholecystitis	**69.29**	67.28	64.51	61.57	65.59	64.66
Diverticulitis	**48.64**	**48.64**	41.63	47.08	46.69	47.86
Pancreatitis	64.13	64.13	**65.80**	65.06	65.43	60.22
Mean	**69.81**	69.41	67.23	67.67	68.83	67.53

Table 4: **OASST diagnostic performance with different diagnostic information orderings.** Changing the order of the presented information changes diagnostic accuracy. Crucially, the best order (bold) is disease specific, meaning a clinician must deliver a preliminary diagnosis to ensure proper model performance, eliminating many of the benefits of an AI clinical decision making system.

Experiment<br>Pathology	Imaging, Lab<br>Physical	Imaging, Physical<br>Lab	Lab, Imaging<br>Physical	Lab, Physical<br>Imaging	Physical, Imaging<br>Lab	Physical, Lab<br>Imaging
Appendicitis	96.97	96.76	96.55	96.34	**97.18**	96.66
Cholecystitis	40.74	37.19	41.82	37.96	36.42	**51.39**
Diverticulitis	51.36	52.53	**54.09**	53.70	53.70	52.92
Pancreatitis	**78.81**	77.88	75.84	75.28	78.07	69.14
Mean	66.97	66.09	67.07	65.82	66.34	**67.53**

Table 5: **WizardLM diagnostic performance with different diagnostic information orderings.** Changing the order of the presented information changes diagnostic accuracy. Crucially, the best order (bold) is disease specific, meaning a clinician must deliver a preliminary diagnosis to ensure proper model performance, eliminating many of the benefits of an AI clinical decision making system.

F Removing Summarization Degrades CDM Diagnostic Accuracy

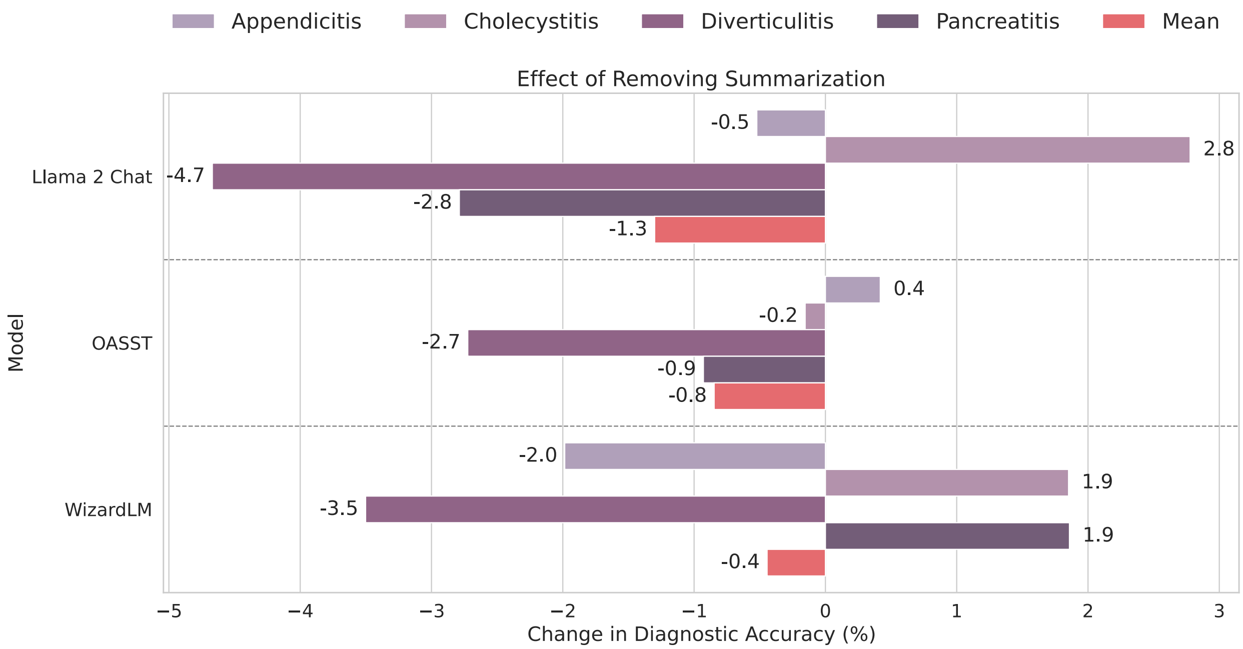

Figure 2: **Not summarizing progress degrades LLM diagnostic accuracy on MIMIC-CDM.** When an LLM approaches its input limit, we ask it to summarize the information gathered thus far to allow for more context. Increased input sizes allows it more opportunities to ask for information and increases the chances of requesting information that is important for the diagnosis.

G Diagnosis Definitions

Pathology	Location	Modifiers
appendicitis	appendi	gangren<br>infect<br>inflam<br>abscess<br>rupture<br>necros<br>perf
cholecystitis	gallbladder	gangren<br>infect<br>inflam<br>abscess<br>necros<br>perf
cholecystitis	cholangitis	cholangitis
cholecystitis	acute gallbladder	disease<br>attack
cholecystitis	acute biliary	colic
diverticulitis	diverticul	inflam<br>infect<br>abscess<br>perf<br>rupture
diverticulitis	acute colonic	perfor
diverticulitis	sigmoid	perfor<br>colitis
pancreatitis	pancrea	gangren<br>infect<br>inflam<br>abscess<br>necros

Table 6: **Accepted diagnostic phrasings for each pathology.** For each pathology, alternative diagnostic phrasings were accepted. If within one diagnostic phrase (delineated through e.g. periods, commas, 'vs', or other separators) the location and modifier occurred without negation, the diagnosis was marked as correct. For example, for cholecystitis a diagnosis of 'Perforated gallbladder' or 'Gallbladder infection' were both accepted. A diagnosis of 'Gallbladder disease vs perforated appendix' would not be accepted. Shortened substrings of the locations and modifiers were used to allow for alternative endings to match such as both 'An *infect* of the gallbladder' and '*Infect*ed gallbladder', or 'Peri*appendi*cular abscess' and 'Perforated *appendi*x'. Manual controls were done to verify the specificity and sensitivity of the definitions.

H Dataset Statistics

Disease	Median Age	Age Range	Gender Distribution	Race Distribution
Appendicitis	36	18-95	F (48.8%), M (51.2%)	WHITE (64.9%)<br>BLACK (10.3%)<br>HISPANIC (8.3%)<br>ASIAN (8.0%)<br>OTHER (8.5%)
Cholecystitis	59	18-95	F (55.8%), M (44.2%)	WHITE (63.8%)<br>BLACK (15.1%)<br>HISPANIC (9.2%)<br>ASIAN (5.4%)<br>OTHER (6.5%)
Pancreatitis	59	18-93	F (55.1%), M (44.9%)	WHITE (65.1%)<br>BLACK (15.3%)<br>HISPANIC (8.9%)<br>ASIAN (2.6%)<br>OTHER (8.1%)
Diverticulitis	60	18-91	F (58.9%), M (41.1%)	WHITE (72.1%)<br>BLACK (10.5%)<br>HISPANIC (5.8%)<br>ASIAN (4.7%)<br>OTHER (7.0%)

Table 7: **Demographic Statistics of Patients with Different Diseases**

Disease	Median Age	Age Range	Gender Distribution	Race Distribution
Appendicitis	39	20-75	F (45.0%), M (55.0%)	WHITE (65.0%)<br>BLACK (15.0%)<br>HISPANIC (5.0%)<br>ASIAN (5.0%)<br>OTHER (10.0%)
Cholecystitis	67	31-85	F (45.0%), M (55.0%)	WHITE (65.0%)<br>BLACK (10.0%)<br>HISPANIC (5.0%)<br>ASIAN (5.0%)<br>OTHER (15.0%)
Pancreatitis	53	20-82	F (60.0%), M (40.0%)	WHITE (50.0%)<br>BLACK (10.0%)<br>HISPANIC (15.0%)<br>ASIAN (0.0%)<br>OTHER (25.0%)
Diverticulitis	56	31-85	F (45.0%), M (55.0%)	WHITE (75.0%)<br>BLACK (15.0%)<br>HISPANIC (0.0%)<br>ASIAN (5.0%)<br>OTHER (5.0%)

Table 8: **Demographic Statistics of Physician Comparison Subset of 80 patients**

I LLMs Diagnostic Accuracy Without Medical Abbreviations

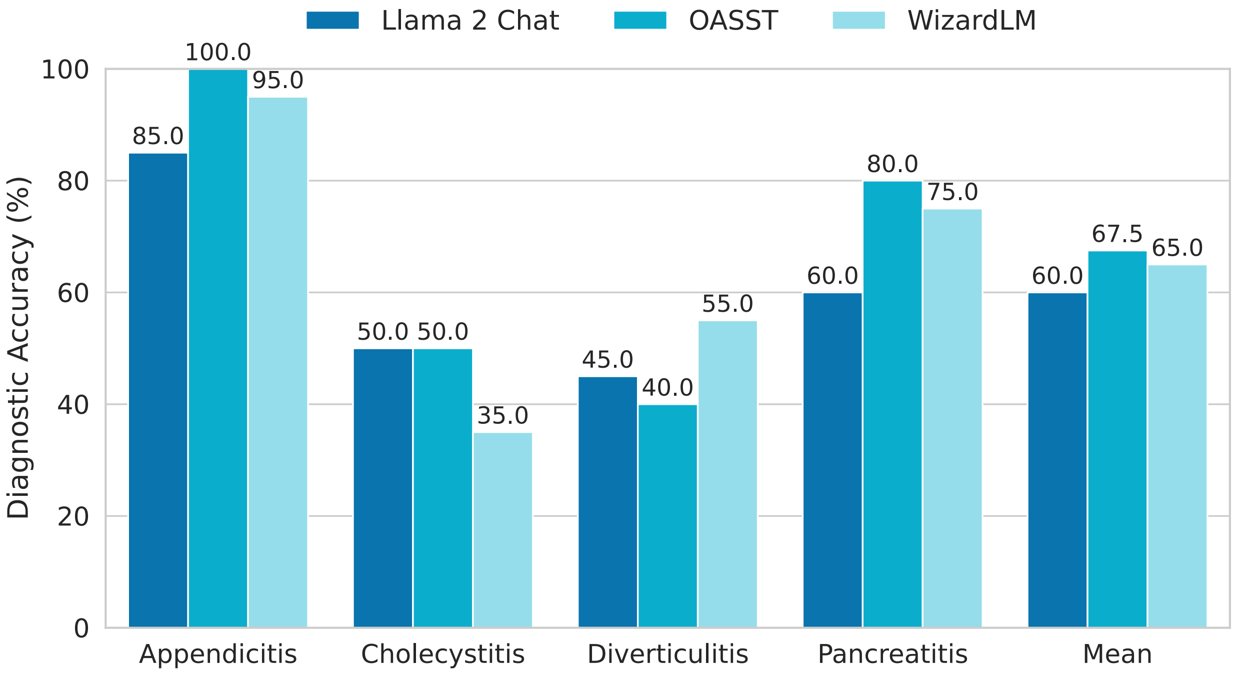

Figure 3: **Expanding medical abbreviations does not improve LLM performance.** Diagnostic accuracy on the clinician subset of MIMIC-CDM-FI stays the same or degrades when medical abbreviations are written out.

J Example Exchange using Synthetic Data

In this section we show an example exchange between our simulated clinical environment provided by our evaluation framework using the MIMIC-CDM dataset and the WizardLM model. The first prompt is the system prompt given by the framework to the model explaining the task and its options. The rest of the exchange pulls (synthetic) data from MIMIC-CDM and records the LLM's response. Seen in this exchange is an example of the model requesting an imaging modality that is not available in the dataset and also hallucinating a surgery tool that does not exist. Upon reminding it of the available tools it correctly diagnoses the patient. This example exchange uses synthetic data to further protect patient privacy.

Evaluation Framework using MIMIC-CDM

You are a medical artificial intelligence assistant. You give helpful, detailed and factually correct answers to the doctors questions to help him in his clinical duties. Your goal is to correctly diagnose the patient and provide treatment advice. You will consider information about a patient and provide a final diagnosis.

You can only respond with a single complete

Thought:
Action:
Action Input:

format OR a single

Thought:
Final Diagnosis:
Treatment:

format. Keep all reasoning in the Thought section. The Action, Action Input, Final Diagnosis, and Treatment sections should be direct and to the point. The results of the action will be returned directly after the Action Input field in the "Observation:" field.

Format 1:

Thought: (reflect on your progress and decide what to do next)
Action: (the action name, should be one of [Physical Examination, Laboratory Tests, Imaging])
Action Input: (the input string to the action)
Observation: (the observation from the action will be returned here)

OR

Format 2:

Thought: (reflect on the gathered information and explain the reasoning for the final diagnosis)
Final Diagnosis: (the final diagnosis to the original case)
Treatment: (the treatment for the given diagnosis)

The tools you can use are:

Physical Examination: Perform physical examination of patient and receive the observations.
Laboratory Tests: Run specific laboratory tests and receive their values. The specific tests must be specified in the 'Action Input' field.
Imaging: Do specific imaging scans and receive the radiologist report. Scan region AND modality must be specified in the 'Action Input' field. Consider the following case and come to a final diagnosis and treatment by thinking, planning, and using the aforementioned tools and format.

Evaluation Framework using MIMIC-CDM

Patient History:
___ presenting with 3 days since acute onset of pain in RLQ. Pain started ___ afternoon but worsened significantly overnight. Intense episodes woke her from sleep. Patient proceeds to experience nausea and chills. No vomiting. Patient endorses anorexia. Past Medical History: Anorexia, Anxiety. Family History: Non-contributory.

LLM

Thought: Given the acute onset of right lower quadrant pain, along with anorexia and right-sided flank pain, there could be several possibilities such as appendicitis, kidney stones, or inflammatory bowel disease like Crohn's disease. However, since the patient does not have any past medical history or family history of these conditions, it might be less likely. Therefore, we need to perform further investigations to determine the exact cause of the pain.
Action: Physical Examination
Action Input: N/A

Evaluation Framework using MIMIC-CDM

Observation: Physical Examination:
Temp: 97.8 HR: 44 BP: 104/69 RR: 17 100% Ra Gen: NAD HEENT: No scleral icterus, mucus membranes moist CV: RRR no m,r,g RESP: CTABL Abd: soft, tender to palpation inRLQ, non distended, no rebound or guarding Ext: wwpx4, palpable distal pulses

LLM

Thought: After performing the physical examination, the vital signs appear normal except for a slightly elevated blood pressure. Findings of tenderness to palpation in the right lower quadrant of the abdomen supports the forementioned possibilities of the patient's complaints, such as appendicitis, kidney stones, urinary tract infection, ovarial cysts or inflammatory bowel disease. We need to investigate further to rule out potential causes.

Action: Laboratory Tests
Action Input: Complete Blood Count (CBC), Liver Function Tests (LFTs), Renal panel, Urinalysis

Evaluation Framework using MIMIC-CDM

Observation: Laboratory Tests:
(Blood) Red Blood Cells: 4.39 m/uL
(Blood) White Blood Cells : 16.8 K/uL
(Blood) Hemoglobin: 12.4 g/dL
(Blood) Hematocrit: 38.8 %
(Blood) MCV: 85.4 fL
(Blood) MCH: 27.3 pg
(Blood) MCHC: 33.4 %
(Blood) Platelet Count: 226.8 K/uL
(Blood) Lymphocytes: 22.0 %
(Blood) Basophils: 0.5 %
(Blood) Eosinophils: 1.9 %
(Blood) Monocytes: 5.5 %
(Blood) Neutrophils: 69.8 %
(Blood) RDW: 13.0 %
(Blood) Alanine Aminotransferase (ALT): 18.1 IU/L
(Blood) Asparate Aminotransferase (AST): 29.1 IU/L
(Blood) Alkaline Phosphatase: 68.8 IU/L
(Blood) Bilirubin, Total: 0.3 mg/dL
(Blood) PT: 10.5 sec
(Blood) INR(PT): 0.9
(Blood) Albumin: 5.2 g/dL
(Blood) Creatinine: 0.6 mg/dl
(Urine) Ketones: NEG
(Urine) Urine Color: Yellow.
(Urine) Urine Appearance: Clear.
(Urine) Specific Gravity: 1.0
(Urine) Protein: NEG.
(Urine) pH: 5.8 units
(Urine) Bilirubin: NEG.
(Urine) Glucose: NEG.
(Urine) Urobilinogen: NEG.
(Urine) Ketone: TR.
(Urine) Nitrite: NEG.
(Urine) Leukocytes: NEG.

LLM

Thought: The laboratory test results show normal values for most parameters analyzed. However, the patients has elevated white blood cells as a marker for inflammation. Based on the diagnostic test results so far, the patient is suspected to have an inflammation in the abdomen, such as appendicitis or inflammatory bowel disease.

Action: Imaging
Action Input: Ultrasound of the Right Lower Quadrant

Evaluation Framework using MIMIC-CDM

Observation: Imaging:
Abdomen Ultrasound: Not available. Try a different imaging modality.

LLM

Thought: Since ultrasound is not suitable for this case, we should proceed with another imaging modality. Given the possibility of hepatobiliary involvement, a computed tomography (CT) scan would be more appropriate to visualize the internal organs better.

Action: Imaging
Action Input: Computed Tomography (CT) scan of the Abdom

Evaluation Framework using MIMIC-CDM

Observation: Imaging:
Abdomen CT: EXAMINATION:
CT ABD AND PELVIS WITH CONTRAST:

TECHNIQUE:
MDCT imaging, extending from the lung bases to the pubic symphysis, was performed post-administration of 130cc intravenous contrast. Axial scans were analyzed alongside coronal and sagittal reformatted images.

FINDINGS:

CHEST:
Lung bases are clear without pleural or pericardial effusions.

ABDOMEN:
Liver and gallbladder present normally; portal and hepatic veins are patent. Pancreas and spleen are normal; bilateral adrenal glands are unremarkable. Kidneys show symmetrical enhancement without hydronephrosis.

Stomach and small bowel maintain normal caliber. A hyperdense, dilated structure, likely the appendix, arises from the cecal base, measuring up to 10 mm and exhibiting mucosal hyperenhancement compared to adjacent bowel. Predominant features include hyperenhancement and wall thickening, with less emphasis on fluid content. Inflammatory changes are evident at the cecal base, around the appendix, especially at its indistinct tip.

Adjacent to the ascending colon's mesentery, two enlarged lymph nodes, 1.5 cm and 1.9 cm in size, show brisk enhancement. The smaller node contains central low-density material. Both nodes are surrounded by marked fat stranding and suspected necrotic or suppurative changes.

The ascending, transverse, and descending colon appear normal. No mesenteric free air is observed.

PELVIS:
Urinary bladder and adnexa are unremarkable. Uterus contains an intrauterine device in standard position. A small volume of simple free fluid is present in the pelvis, likely physiologic.

VESSELS:
Aorta and its major branches are normal in caliber and patent.

OSSEOUS STRUCTURES:
No focal lytic or sclerotic lesions suggestive of malignancy are identified.

LLM

Thought: The CT scan supports the diagnosis of acute appendicitis due to the enlarged and inflamed appendix observed. I will recommend surgical intervention for removal of the affected organ.
Action: Surgery
Action Input: Appendectomy

Evaluation Framework using MIMIC-CDM

Observation: Provide a diagnosis and treatment OR a valid tool. That is not a valid tool, try one of [Physical Examination, Laboratory Tests, Imaging].

LLM

Thought:
The CT scan revealed an inflamed and enlarged appendix, indicating acute appendicitis as the most likely cause of the patient's pain. Additionally, there are two enlarged and briskly enhancing lymph nodes in the mesentery, which could suggest a secondary infectious process or inflammatory condition. The presence of a small amount of simple free fluid in the pelvis might be due to the inflammatory response.

Final Diagnosis: Acute Appendicitis
Treatment: Surgical intervention - Appendectomy

K Investigating Additional Metrics

Model	Pathology	Sensitivity	Specificity	F1	PPV	NPV
Llama 2 Chat	appendicitis	95.1	92.5	93.8	92.0	95.4
	cholecystitis	48.1	93.7	63.6	77.2	80.2
	diverticulitis	44.0	99.5	61.0	93.4	92.5
	pancreatitis	48.0	87.9	62.0	56.8	83.5
OASST	appendicitis	96.9	95.4	96.1	94.9	97.2
	cholecystitis	61.4	95.6	74.8	85.6	85.3
	diverticulitis	48.2	99.8	65.1	97.6	93.3
	pancreatitis	64.5	93.9	76.5	78.2	88.7
WizardLM	appendicitis	96.5	96.8	96.6	96.5	96.8
	cholecystitis	37.4	97.2	54.0	84.9	78.8
	diverticulitis	52.9	99.6	69.1	95.1	93.8
	pancreatitis	73.8	85.7	79.3	63.8	90.5

Table 9: **Additional metrics.** This table shows additional performance metrics calculated on the MIMIC-CDM-FI dataset for the three primary models investigated in this study. Sensitivity is equal to per-class accuracy used throughout this work. To calculate these additional metrics, false positives and true negatives were counted over the diseases included in this dataset which is not representative of a general population and the metrics should thus be interpreted with caution.
