# Supplementary material for: Evaluation and mitigation of the limitations of large language models in clinical decision-making
Source: Nat Med. 2024 Jul 4;30(9):2613–22. doi: 10.1038/s41591-024-03097-1 (PMC11405275; doi:10.1038/s41591-024-03097-1)
Supplement: Supplementary file 2 — Reporting Summary [file 41591_2024_3097_MOESM2_ESM.pdf]

nature portfolio

Corresponding author(s): Paul Hager

Last updated by author(s): Apr 3, 2024

Reporting Summary

Nature Portfolio wishes to improve the reproducibility of the work that we publish. This form provides structure for consistency and transparency in reporting. For further information on Nature Portfolio policies, see our Editorial Policies and the Editorial Policy Checklist.

Statistics

For all statistical analyses, confirm that the following items are present in the figure legend, table legend, main text, or Methods section.

n/a	Confirmed	
☐	☒	The exact sample size (n) for each experimental group/condition, given as a discrete number and unit of measurement
☐	☒	A statement on whether measurements were taken from distinct samples or whether the same sample was measured repeatedly
☐	☒	The statistical test(s) used AND whether they are one- or two-sided<br>*Only common tests should be described solely by name; describe more complex techniques in the Methods section.*
☐	☒	A description of all covariates tested
☐	☒	A description of any assumptions or corrections, such as tests of normality and adjustment for multiple comparisons
☐	☒	A full description of the statistical parameters including central tendency (e.g. means) or other basic estimates (e.g. regression coefficient) AND variation (e.g. standard deviation) or associated estimates of uncertainty (e.g. confidence intervals)
☐	☒	For null hypothesis testing, the test statistic (e.g. F, t, r) with confidence intervals, effect sizes, degrees of freedom and P value noted<br>*Give P values as exact values whenever suitable.*
☒	☐	For Bayesian analysis, information on the choice of priors and Markov chain Monte Carlo settings
☒	☐	For hierarchical and complex designs, identification of the appropriate level for tests and full reporting of outcomes
☒	☐	Estimates of effect sizes (e.g. Cohen's d, Pearson's r), indicating how they were calculated

Our web collection on statistics for biologists contains articles on many of the points above.

Software and code

Policy information about availability of computer code

Data collection	The code used to create the dataset can be found at: https://github.com/paulhager/MIMIC-Clinical-Decision-Making-Dataset<br>The code to create the dataset uses python v3.10 and pandas v2.1.3.
Data analysis	The evaluation framework used for this study can be found at: https://github.com/paulhager/MIMIC-Clinical-Decision-Making-Framework.<br>The analysis framework to evaluate all results, generate all plots and do all statistical analysis can be found at: https://github.com/paulhager/MIMIC-Clinical-Decision-Making-Analysis.<br>All code uses python v3.10, pytorch v2.1.1, transformers v4.35.2, spacy v3.4.4, langchain v0.0.339, optimum v1.14, thefuzz v0.20, exllamav2 v0.0.8, nltk v3.8.1, negspacy v1.0.4, scispacy v0.5.2, spacy v3.4.4

For manuscripts utilizing custom algorithms or software that are central to the research but not yet described in published literature, software must be made available to editors and reviewers. We strongly encourage code deposition in a community repository (e.g. GitHub). See the Nature Portfolio guidelines for submitting code & software for further information.

Data

Policy information about availability of data

All manuscripts must include a data availability statement. This statement should provide the following information, where applicable:

- Accession codes, unique identifiers, or web links for publicly available datasets
- A description of any restrictions on data availability
- For clinical datasets or third party data, please ensure that the statement adheres to our policy

The dataset is available to all researchers who create an account on https://physionet.org/ and follow the steps to gain access to the MIMIC-IV database (https://physionet.org/content/mimiciv/2.2/). Access is given after completing the "CITI Data or Specimens Only Research" training course. The data use agreement of physionet for "credentialed health data" must also be signed.

The generated dataset can also be directly downloaded from physionet once the appropriate credentials have been gained (see above) under the link: https://physionet.org/content/mimic-iv-ext-cdm/1.0/

Human research participants

Policy information about studies involving human research participants and Sex and Gender in Research.

Reporting on sex and gender	As data was de-identified, no sex or gender based analysis was done for this study.
Population characteristics	As data was de-identified, no population characteristics were considered during this study.
Recruitment	No participants were recruited by the authors for this study.
Ethics oversight	The data used was taken from the MIMIC database which is managed by MIT. As per the MIMIC Website:<br><br>"The collection of patient information and creation of the research resource was reviewed by the Institutional Review Board at the Beth Israel Deaconess Medical Center, who granted a waiver of informed consent and approved the data sharing initiative."

Note that full information on the approval of the study protocol must also be provided in the manuscript.

Field-specific reporting

Please select the one below that is the best fit for your research. If you are not sure, read the appropriate sections before making your selection.

☒ Life sciences ☐ Behavioural & social sciences ☐ Ecological, evolutionary & environmental sciences

For a reference copy of the document with all sections, see nature.com/documents/nr-reporting-summary-flat.pdf

Life sciences study design

All studies must disclose on these points even when the disclosure is negative.

Sample size	The final sample size (2400) was not predetermined and includes all possible samples from the MIMIC database that follow the filter criteria (see below).
Data exclusions	Data was excluded if in the final discharge summary diagnosis of the patient, the first diagnosis written did not include one of the pre-selected pathologies. This was pre-set before study begin to filter for cases that can be primarily attributed to one of the studied pathologies. The pathologies were selected as they are important endpoints of a common initial complaint (abdominal pain) with different diagnostic signals and treatments required.<br><br>Further data was excluded if one of the diagnostic modalities was missing, including history of present illness, physical examination, laboratory events, and radiology reports. This was done as we wanted to test the information gathering capabilities of the models and provide them with information upon request.
Replication	We confirm that all experimental results can be reproduced with the code and data provided.
Randomization	No experimental groups were created for this study as no model training was done. All data were used for evaluation.
Blinding	As there was no group allocation, blinding was not relevant.

Reporting for specific materials, systems and methods

We require information from authors about some types of materials, experimental systems and methods used in many studies. Here, indicate whether each material, system or method listed is relevant to your study. If you are not sure if a list item applies to your research, read the appropriate section before selecting a response.

Materials & experimental systems

n/a	Involved in the study
☒	☐ Antibodies
☒	☐ Eukaryotic cell lines
☒	☐ Palaeontology and archaeology
☒	☐ Animals and other organisms
☒	☐ Clinical data
☒	☐ Dual use research of concern

Methods

n/a	Involved in the study
☒	☐ ChIP-seq
☒	☐ Flow cytometry
☒	☐ MRI-based neuroimaging
